# Respiratory metabolism and calorie restriction relieve persistent endoplasmic reticulum stress induced by calcium shortage in yeast

**DOI:** 10.1038/srep27942

**Published:** 2016-06-16

**Authors:** Stefano Busti, Valeria Mapelli, Farida Tripodi, Rossella Sanvito, Fulvio Magni, Paola Coccetti, Marcella Rocchetti, Jens Nielsen, Lilia Alberghina, Marco Vanoni

**Affiliations:** 1 Department of Biotechnology and Biosciences, University of Milano-Bicocca, Milan, Italy; 2 SYSBIO, Centre of Systems Biology, Milan, Italy; 3 Department of Biology and Biological Engineering, Division of Industrial Biotechnology, Chalmers University of Technology, Gothenburg, Sweden; 4 Department of Health Sciences, University of Milano-Bicocca, Milan, Italy; 5 Department of Biology and Biological Engineering, Chalmers University of Technology, Gothenburg, Sweden; 6 Novo Nordisk Foundation Center for Biosustainability, Technical University of Denmark, Hørsholm, Denmark

## Abstract

Calcium homeostasis is crucial to eukaryotic cell survival. By acting as an enzyme cofactor and a second messenger in several signal transduction pathways, the calcium ion controls many essential biological processes. Inside the endoplasmic reticulum (ER) calcium concentration is carefully regulated to safeguard the correct folding and processing of secretory proteins. By using the model organism *Saccharomyces cerevisiae* we show that calcium shortage leads to a slowdown of cell growth and metabolism. Accumulation of unfolded proteins within the calcium-depleted lumen of the endoplasmic reticulum (ER stress) triggers the unfolded protein response (UPR) and generates a state of oxidative stress that decreases cell viability. These effects are severe during growth on rapidly fermentable carbon sources and can be mitigated by decreasing the protein synthesis rate or by inducing cellular respiration. Calcium homeostasis, protein biosynthesis and the unfolded protein response are tightly intertwined and the consequences of facing calcium starvation are determined by whether cellular energy production is balanced with demands for anabolic functions. Our findings confirm that the connections linking disturbance of ER calcium equilibrium to ER stress and UPR signaling are evolutionary conserved and highlight the crucial role of metabolism in modulating the effects induced by calcium shortage.

Calcium regulates a wide variety of cellular processes by acting as an enzyme cofactor and a second messenger in several signal transduction pathways. Intracellular ion homeostasis and a precise regulation of calcium-triggered signaling mechanisms are therefore crucial to the survival of all organisms^1^,^2^,^3^.

Like all eukaryotes, *Saccharomyces cerevisiae* typically maintains free cytosolic Ca^2+^ concentration extremely low, within a sub-micromolar range (50–200 nM), whereas the total cellular content is 10000-fold higher (2–3 mM)^3^. The vacuole is the primary storage site for calcium in yeast (>90% of total) and maintains the cytosolic levels of the ion within a narrow physiological range compatible with cell viability: excess calcium is removed from the cytosol by the vacuolar Ca^2+^/ATPase Pmc1 and by the H^+^/Ca^2+^ antiporter Vcx1^3^,^4^.

Calcium levels within the lumen of the endoplasmic reticulum (ER) and Golgi apparatus are carefully regulated by the Ca^2+^/ATPases Spf1 and Pmr1^3^ to ensure the retention of resident luminal proteins and the proper folding and processing of proteins that transit through the secretory pathway^5^. Depletion of calcium ions from the ER by chelators affects the efficiency of protein folding inside the organelle (ER stress) and triggers the unfolded protein response (UPR)^6^, a highly conserved signaling network devoted to restore ER homeostasis^7^,^8^. UPR induction alleviates ER stress and promotes cell survival by increasing the transcription of genes required for protein folding and degradation, ER expansion and secretory trafficking. However, when ER dysfunctions are severe and persistent, a prolonged activation of the UPR signaling can trigger a cell death program by enhancing ROS (reactive oxygen species) accumulation^9^,^10^,^11^,^12^,^13^,^14^,^15^.

Diverse physiological conditions elicit a rapid, transient increase in the cytosolic calcium level, either by promoting ion influx from the external medium or by releasing it from internal stores^3^. In yeast, calcium signals are generated during mating, after exposure to certain environmental stresses (such as osmotic shock, ionic stress, ER stress, oxidative stress, high temperature, alkaline pH, several antifungal drugs), after glucose addition to starved-cells and during mitosis^3^,^16^. Other processes affected by calcium include actin cytoskeleton organization and vacuolar fusion.

In contrast to the wide knowledge of the physiological conditions that trigger temporal and spatial increase in calcium level, little is known about the effects of calcium shortage in *S. cerevisiae*. Yeast cells appear to grow indefinitely in calcium-deficient medium^17^, presumably thriving on residual ion contamination. However, by using chelators and ionophores, it was shown that calcium depletion causes a transient G1 arrest, followed by a G2/M block^17^. The study suggested that Ca^2+^ is essential for all stages of the cell cycle (except DNA synthesis) and that can positively control G1 events by regulating the intracellular cAMP level^17^. These results were later questioned, since manganese addition to calcium-depleted media effectively supports cell-cycle progression^18^. Nonetheless, the existence of a global transcriptional remodeling induced by low calcium levels in yeast was confirmed by microarray technology^19^.

Here we report that calcium shortage enhances ROS production, slows down growth and metabolism and induces cell death. The oxidative stress in calcium-starved cells may result from accumulation of unfolded proteins within the lumen of the endoplasmic reticulum (ER stress). The effects of calcium shortage are severe during growth on fermentable sources and can be rescued by reducing protein synthesis or by stimulating cellular respiration, suggesting that the energetic and macromolecular metabolism can ultimately control the fate of calcium-starved yeast cells.

## Results

### Calcium shortage decreases growth rate and cell viability

**Figure 1:**
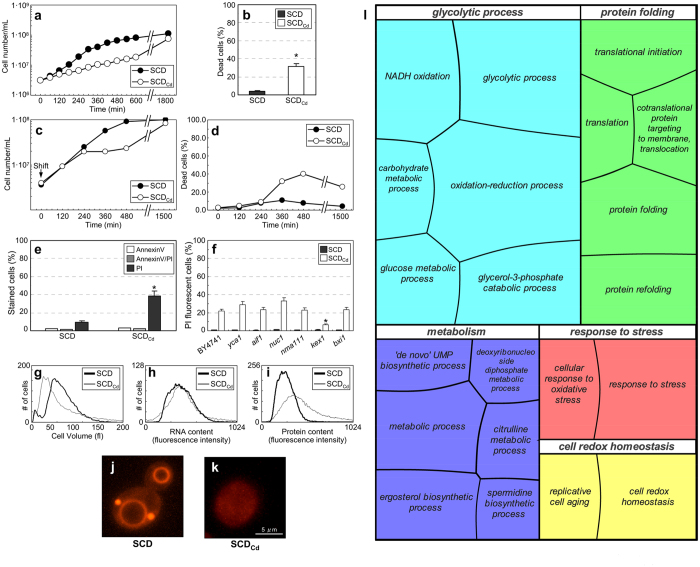
Calcium shortage affects cell growth, cell viability and vacuolar morphology. (**a**) W303-1A cells were grown at 30 °C in SC and SC_Cd_ liquid medium supplemented with 2% (w/v) glucose. A representative experiment out of ten performed is shown. (**b**) Fraction of dead cells in logarithmic phase cultures in SCD and SCD_Cd_ glucose media, as evaluated by direct microscopic examination after trypan blue staining (and confirmed by cytofluorimetric analysis). Means ± Standard Deviations (SDs) of five biological replicates are shown (n > 1000 cells; *p < 0.05, two-tailed *t*-test). (**c,d**) Cells were cultivated to log-phase in SCD medium. At time point 0, the culture was split and cells were resuspended either in SCD or SCD_Cd_ fresh media. Cellular densities of the cultures (**c**) and cell viability (**d**) are shown. A representative experiment out of three performed is shown. (**e**) Phosphatidylserine externalization and loss of membrane integrity in cells exponentially growing in SCD or SCD_Cd_ media detected by cytofluorimetric analysis after Annexin V/propidium iodide co-staining. Means ± SDs of three biological replicates are shown (*p <  < 0.05, two-tailed *t*-test). (**f**) Mutants defective in the apoptotic program were cultivated to late exponential phase in SCD or SCD_Cd_ media. Cell viability was evaluated by cytofluorimetric analysis. Means ± SDs of two biological replicates are shown (*p < 0.05, two-tailed *t*-test). (**g–i**) Cell volume distributions obtained by Coulter analysis (**g**) and protein and RNA distribution profiles obtained by cytofluorimetric analysis (**h,i**) for cells exponentially growing in SCD or SCD_Cd_ media. (**j,k**) Vacuole morphology in cells exponentially growing in SCD or SCD_Cd_ media stained with FM4-64. Representative fluorescence microscopy images are shown. (**l**) Comparative proteome analysis performed on cells exponentially growing in SCD or SCD_Cd_ media. Data are visualized as a treemap, in which related Gene Ontology Biological Process terms are joined into loosely related superclusters. Size of the tiles reflects the *p*-value.

Growth in calcium-depleted medium supplemented with 2% (w/v) glucose (SCD_Cd_) induced a ~2.5 fold increase in mass duplication time (MDT), without altering the budding index, in comparison with cultures in medium containing regular calcium concentration (SCD) (Fig. 1a; Table SI; Fig. S1a–c).

About one-third of the cells grown in SCD_Cd_ medium were unviable (Fig. 1b), the fraction of dead cells being strain-dependent (Fig. S1d). Transfer of SCD-growing cells to SCD_Cd_ medium reduced the proliferation rate starting from 240 min after the shift and a significant drop in cell viability was also evident at 360 min (Fig. 1c,d). Eventually, cells adapted to calcium shortage and the fraction of dead cells stabilized at ~30% (Fig. 1c,d). Cell death was accompanied by an increase in the proportion of cells stained with propidium iodide (PI), which is indicative of plasma membrane rupture (a cytological hallmark of “primary necrosis”; Fig. 1e). Apoptosis was apparently not induced, since the fraction of cells positive to Annexin V staining was negligible and none of the tested mutants defective in the apoptotic program (except *kex1*) exhibited a survival advantage when exposed to calcium shortage (Fig. 1e,f).

SCD_Cd_-grown cells showed a ~25% decrease in their mean volume, which was not accompanied by a parallel decrease in the average protein and RNA content (Fig. 1g–i; Table SI; Fig. S2a). Vacuole-specific labeling with either FM4-64 or CDCFDA (two fluorescent dyes with complementary staining pattern) failed to detect a proper organelle structure in most SCD_Cd_-grown cells, which instead exhibited diffuse staining and irregular fluorescent patches, (Fig. 1j,k; S2b–g) that were not simply due to defects in the dye internalization or to cell death (Fig. S2h–o), but were rather consistent with the presence of small, unstructured acidic vescicles. The reduced size of calcium-starved cells may thus derive from defects in vacuolar biogenesis and/or vesicular trafficking, a phenotype associated with necrotic death^12^,^20^,^21^. However, no effect on cell survival under calcium shortage was detected in strains with impaired vacuolar function, including the *vma1* mutant, which lacks the V-ATPase (vacuolar H^+^-ATPase) known to trigger necrosis in calcineurin-deficient cells treated with ER stressors^12^ (Fig. S2p–q).

To get a system-level overview of pathways and functions affected by calcium shortage we performed differential proteomics analysis on cells cultivated in SCD and SCD_Cd_ media. The expression level of ~10% of the proteins visualized on 2D-PAGE gels was modulated by calcium (Table SII; Fig S3). Gene Onthology (GO) terms enriched in calcium-modulated proteins are shown as a hierarchical “treemap”^22^ (Fig. 1l) that indicates metabolism, oxidative stress and protein folding as major functions affected by calcium depletion.

### Calcium shortage induces a nutritionally-modulated metabolic reprogramming

**Figure 2:**
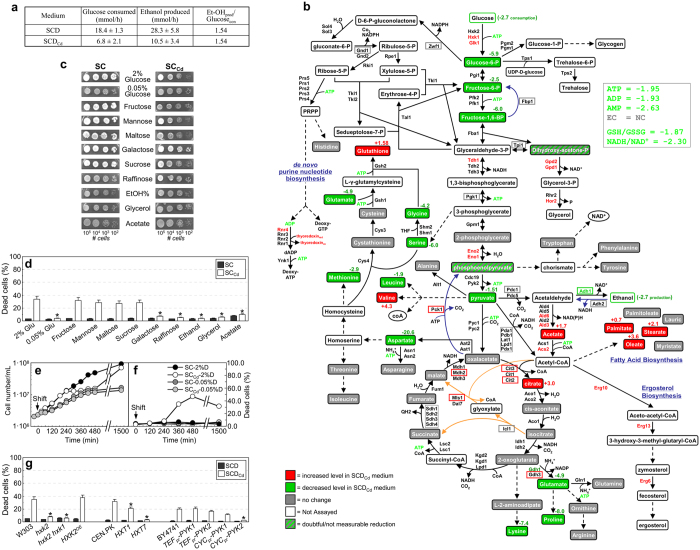
The effects of calcium shortage are carbon source-dependent. (**a**) Glucose consumption and ethanol production rates were determined in cells grown overnight in either SCD or SCD_Cd_ media and resuspended in medium containing 50 mM glucose at a final density of about 4*10^6 ^cells/mL. A glucose-to-ethanol ratio close to 2 moles of ethanol produced per moles of glucose consumed (the maximum theoretical value) indicates a mostly fermentative metabolism. Values are means ± SDs of three biological replicates. (**b**) Alterations in the proteomic and metabolomic profiles of cells grown under calcium shortage in 2% glucose medium (SCD_Cd_). Changes in protein or mRNA levels were evaluated by 2D-page or qRT-PCR, respectively. Increase/decrease under calcium shortage are red/green colored; grey indicates no significant change. A colored box indicates transcriptional regulation under calcium shortage. Proteins whose expression changes under calcium shortage are colored. Blue lines indicate reactions specific for the gluconeogenic pathway. Orange lines indicate reactions specific for the glyoxylate cycle. (**c**) Cellular suspensions of CEN.PK2-1C cells were serially diluted and spotted on SC and SC_Cd_ media plates supplemented with the indicated carbon sources. (**d**) Cell viability under calcium shortage during exponential growth in liquid media on the indicated carbon source, as evaluated by cytofluorimetric analysis. Values are means ± SDs of two biological replicates (*p < 0.05, two-tailed *t*-test). (**e,f**) Log-phase W303-1A cells cultivated in SCD medium containing 2% (w/v) glucose were shifted in either SC or SC_Cd_ media supplemented with 0.05% (w/v) glucose (calorie restriction). Cell density (**d**) and cell viability (**e**) are shown. A representative experiment out of three performed is shown. (**g**) Cell viability under calcium shortage for mutants with reduced hexokinase, sugar uptake and pyruvate kinase activity, as evaluated by cytofluorimetric analysis. Values are means ± SDs of three biological replicates (*p < 0.05, two-tailed *t*-test).

To study the effect of calcium shortage on metabolism, we first measured glucose utilization and ethanol excretion. Glucose consumption and ethanol production rates were about three times lower in calcium-starved cells (Fig. 2a), proportionally to their decreased growth rate (Fig. 1a; Table SI). Many glycolytic intermediates were also significantly reduced (Table 1; Fig. 2b).

Consistent with the reduction of secreted ethanol, a down-regulation of alcohol dehydrogenase Adh1 was detected in SCD_Cd_-grown cells (Table SII; Fig. 2b), alongside an up-regulation of enzymes involved in glycerol (Gpd1, Gpd2 and Hor2), acetate and acetyl-CoA biosynthesis (Ald6, Acs2 and Ald3) (Fig. 2b; Table SII). Calcium shortage resulted in increased levels of extracellular acetate and intracellular citrate, palmitate, stearate and oleate, as well as in up-regulation of several ER-localized enzymes (Erg10, Erg6 and Erg13) required for ergosterol biosynthesis from acetyl-CoA (Table 2; Fig. 2b; Table SII).

Metabolite assays, combined with expression profiling of genes encoding glycolytic, gluconeogenic and respiratory enzymes suggested that calcium-starved cells adopted a prevalent fermentative metabolism (Table 1, 2, Fig. 2a,b; Fig. S4a). The observed two-fold reduction in ATP level (Table 1; Fig. 2b) may indicate that calcium shortage lowered the efficiency of the energetic metabolism. In addition, several glucose-repressed proteins (e.g. Hxk1, Glk1, Tdh1, Eno1, Ald3, Rnr4 and Suc2 invertase) were up-regulated in SCD_Cd_ medium, suggesting that “glucose repression”^23^ mechanisms were slightly defective in calcium-starved cells (Fig. 2b; Fig. S4a–d; Table SII).

A significant decrease in the intracellular level of most amino acids was also detected, possibly resulting from reduced biosynthesis from glycolytic/Krebs intermediates or faulty transport/storage in vacuole (Table 2; Fig. 2b).

Calcium shortage-related phenotypes (reduced growth rate, small size, decreased viability, alterations in metabolic profile) were mitigated by growing cells in SC_Cd_ media supplemented with poorly (galactose and raffinose) or non-fermentable (ethanol, glycerol and acetate) carbon sources, or with 0.05% glucose (calorie restriction) (Fig. 2c–f; Table 1, 2; Table SI–SIII).

Conversely, cells cultivated in SC_Cd_ medium containing 2% (w/v) fermentable sugars (glucose, fructose, mannose, sucrose (hydrolyzed by extracellular invertase to yield glucose and fructose) or maltose (intracellularly converted in two glucose units)) displayed the typical calcium shortage-related phenotypes (Fig. 2c,d; Table SI). Glucose, fructose and mannose are all substrates for hexokinase and decreasing their utilization by inactivating the hexokinase-encoding genes (*HXK1* and *HXK2*) had a positive impact on growth rate and cell viability in SC_Cd_ medium (Fig. 2g; Table SI).

Both loss of hexokinase and calorie restriction result in activation of cellular respiration in response to the reduced sugar influx into glycolysis^24^,^25^. Accordingly, other mutants with constitutively active mitochondrial respiration^26^,^27^ were also resistant to calcium shortage-induced death (e.g. a strain with impaired glucose uptake capacity, expressing the high-affinity, low-capacity carrier *HXT7* as the sole transporter or the *CYC*_*pr*_*-PYK2* mutant, having an extremely low pyruvate kinase activity; Fig. 2g).

Taken together, these data suggest that under calcium shortage decreased energetic efficiency (i.e. reduced ATP level) combined with limited availability of building blocks (i.e. amino acids) synthesized from glycolytic/Krebs intermediates may be insufficient to sustain the fast growth rate characterizing fermenting yeast cells.

### Calcium shortage up-regulates the oxidative stress response and promotes ROS accumulation

**Figure 3:**
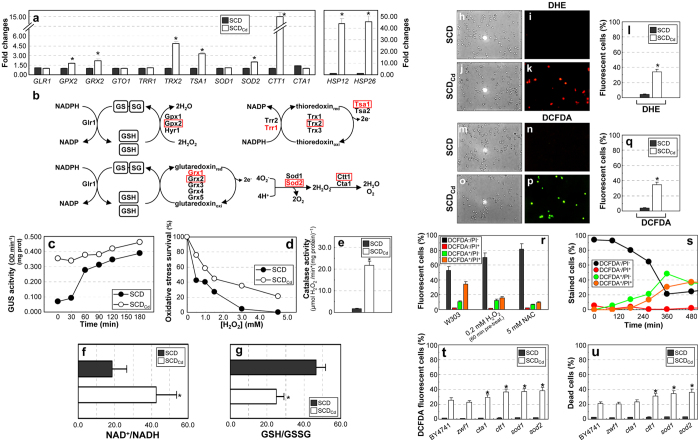
Calcium shortage upregulates the oxidative stress response and promotes ROS accumulation. (**a**) qRT-PCR analysis of transcripts encoding proteins involved in stress response. Values are means ±SDs of biological duplicates (*p < 0.05, two-tailed *t*-test). (**b**) Mechanisms protecting yeast cells against oxidative stress. A red box indicates transcriptional upregulation occurring under calcium shortage (as verified by qRT-PCR). Proteins whose expression levels increases (as confirmed by proteomic analysis) are red colored. (**c**) Log-phase W-GUS cells were exposed to mild oxidative stress (0.5 mM H_2_O_2_) and the time-course activity of the *HSP12::GUS* reporter evaluated. A representative experiment out of three performed is shown. (**d**) SCD- or SCD_Cd_-growing cells were treated 1 hour with H_2_O_2_. Cell survival was evaluated by viable count. A representative experiment out of three performed is shown. (**e**) Catalase activity in cells cultivated in SCD or SCD_Cd_ media. Values are means ±SDs of biological duplicates (*p < 0.05, two-tailed *t*-test). (**f,g**) NAD^+^/NADH and GSH/GSSG ratios in cells grown in SCD or SCD_Cd_ media. Values are means ±SDs of biological duplicates (*p < 0.05, two-tailed *t*-test). (**h–q**) SCD- or SCD_Cd_-grown were stained with either dihydroethidium (DHE, specifically oxidized by superoxide ions to fluorescent ethidium; **h–l**) or dichlorodihydrofluorescein diacetate (DCFDA, oxidized by ROS to fluorescent dichlorofluorescein); **m–q**). Representative microscopic images and the corresponding quantifications are shown. (Means ±SDs of biological triplicates; n > 1000 cells; *p < 0.05, two-tailed *t*-test)). (**r**) Calcium-starved cells were co-stained with DCFDA and propidium iodide (PI) for simultaneous detection of ROS and viability. Where indicated, cells were either pre-treated 1 hour with 0.2 mM H_2_O_2_ before calcium starvation to activate the oxidative-stress response or grown in the presence of the antioxidant NAC (5 mM). Cells were classified according to their fluorescence pattern by direct microscopic observation. Values are means ±SDs of biological duplicates (n > 500 cells). (**s**) SCD-cultivated cells were transferred to SCD_Cd_. Intracellular ROS accumulation and cell viability were evaluated by direct observation of DCFDA/PI co-stained cells. Data representative from biological duplicates are shown. (**t,u**) ROS accumulation (**s**) and cell viability (**t**) in mutants defective in the oxidative stress response (evaluated by cytofluorimetry). Values are means ± SDs of biological duplicates (*p < 0.05, two-tailed *t*-test).

The proteomics analysis (Fig. 1l; Table SII) suggested that mechanisms regulating the cellular response to oxidative stress were constitutively activated in calcium-starved cells. Transcription of genes encoding molecular chaperones and enzymes eliciting defenses against oxidizing agents was significantly up-regulated in SCD_Cd_ -grown cells (Fig. 3a,b). Accordingly, the basal activity of the *HSP12*-*GUS* stress-responsive reporter gene and the resistance to hydrogen peroxide were increased under calcium shortage (Fig. 3c,d). The enhanced resistance to oxidative stress may be largely mediated by the Ctt1 cytosolic catalase that showed strong transcriptional and enzymatic up-regulation (Fig. 3a,e; Table SII). No cross-protection^28^ to other forms of stress (heat-, osmotic or ionic-shock) was detected (Fig. S5).

The existence of an unbalanced oxido-reductive intracellular environment in calcium-starved cells was supported by the shift of both NADH/NAD^+^ and GSH/GSSG ratios towards their oxidized forms (Fig. 3f,g). More than 30% of cells cultivated to mid/late exponential phase in SCD_Cd_ medium showed enhanced production of oxygen radicals (ROS; Fig. 3h–q, Fig. S7a–b).

In order to evaluate if ROS accumulation precedes cell death, cells were double-stained with dichlorodihydrofluorescein diacetate (DCFDA) and propidium iodide (PI). Most of dead cells were positive to ROS staining (DCFDA^+^/PI^+^, orange bar; Fig. S7). A small but sizable fraction of SCD_Cd_-grown cells was positive for ROS staining, but still viable (DCFDA^+^/PI^−^ cells, Fig. 3r, green bar; Fig. S7a–b). Figure 3s reports a shift experiment from SCD to SCD_Cd_ medium. Viable cells with no ROS decrease over time time (PI^−^/DCFDA^−^ cells, black circles). A sub-population of a ROS-producing, viable cells (DCFDA^+^/PI^−^, Fig. 3s, green circles) was detectable 180 min after the shift, preceding the appearance of dead cells positive to ROS staining (DCFDA^+^/PI^+^ cells, orange circles), which became evident only 240 min after the shift. Cytofluorimetric analysis (reported in Fig. S7c) and time-lapse experiments (Fig. S7d and Supplementary Movie 1) points in the same direction. A co-staining with different probes to evaluate ROS production (dihydroethidium (DHE)) and cell viability (Sytox Green) yielded similar results (Fig. S7e–g). Taken together, these data indicate that ROS accumulation precedes cell death in calcium starved yeast cells.

Cells pre-treated with sub-lethal doses of hydrogen peroxide (to activate the oxidative stress response) exhibited a better survival when exposed to calcium shortage (Fig. 3r). Similarly, a treatment with the antioxidant N-acetyl-cysteine^29^,^30^ (NAC) reduced ROS accumulation and significantly improved cell survival (Fig. 3r; Fig S7h), thus suggesting that oxygen radicals play a key role in the death process induced by calcium shortage. Consistently, inactivation of genes encoding enzymes involved in ROS detoxification (*SOD1, SOD2, CTT1* and *CTA1*) further increased oxidative stress and cell death rate under calcium shortage (Fig. 3t,u), whereas wild-type cells cultivated on non-fermentable carbon sources and the *hxt, hxk* and *pyk* mutants did not accumulate oxygen radicals and maintained full viability in calcium-depleted media (S6a–c; Fig. 2d–g).

### Mitochondria are not the main source of intracellular ROS accumulation under calcium shortage

**Figure 4:**
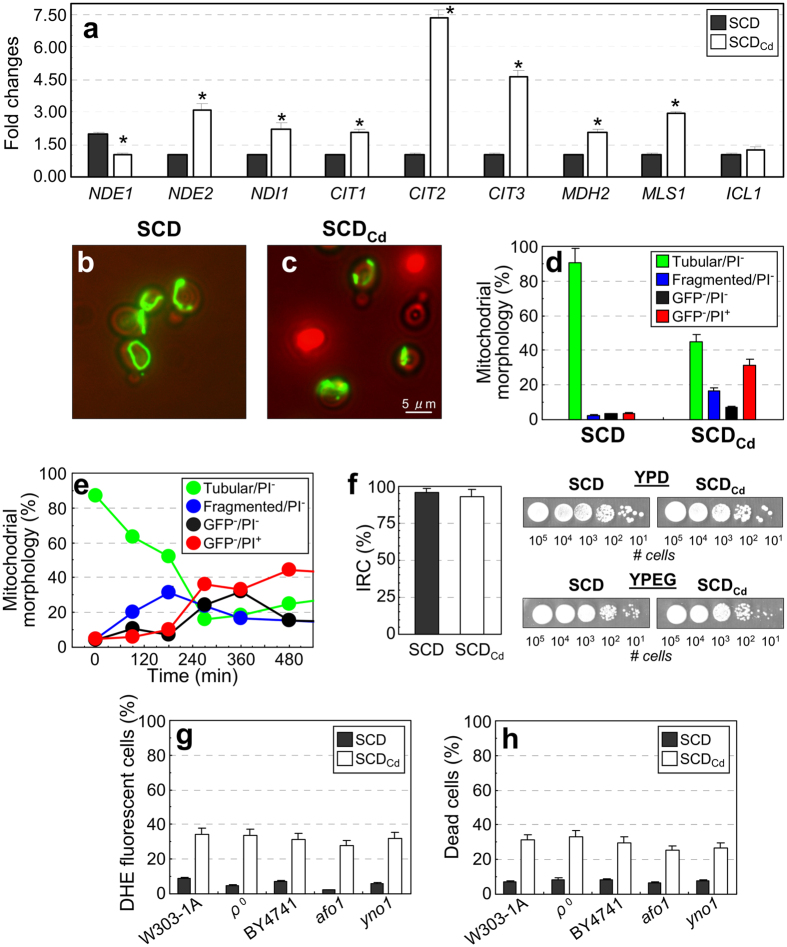
Loss of mitochondrial function does not prevent ROS accumulation under calcium shortage. (**a**) Expression profile of genes encoding for mitochondrial NADH dehydrogenases and enzymes of the glyoxylate cycle under calcium shortage (obtained by qRT-PCR analysis). Values are means ± SDs of two biological replicates (*p < 0.05, two-tailed *t*-test). (**b,c**) Cells expressing a mitochondria-targeted GFP (mtGFP) were cultivated to log-phase in either SCD or SCD_Cd_ media and stained with propidium iodide. Representative fluorescence microscopy images showing mitochondrial morphology in viable and dead cells are reported. (**d**) Classification of yeast cells growing in SCD_Cd_ media according to the morphology of their mitochondria and their viability. The analysis was performed by direct microscopic observation of propidium iodide-stained cells. Values are means ± SDs of three biological replicates. GFP-negative (GFP^−^) cells lacked any detectable mtGFP signal. Values are means ± SDs of three biological replicates (n > 500 cells). (e) mtGFP-expressing cells were cultivated to log-phase in SCD medium, harvested and transferred in SCD_Cd_ media. At the indicated time points samples were collected and stained with propidium iodide. Cells were classified according to their viability and mitochondrial morphology by direct microscopic observation (n > 500 cells). Data representative from biological duplicate are shown. (**f**) Index of Respiratory Competence (IRC) for cells cultivated under calcium shortage. Values are means ± SDs of three biological replicates (*p < 0.05, two-tailed *t*-test). (**g,h**) Intracellular ROS accumulation and cell viability of *bona fide ρ0, afo1* and *yno1* mutant strains and their isogenic wild-type counterparts during late exponential phase growth in SCD or SCD_Cd_ media, as evaluated by cytofluorimetric analysis. Values are means ± SDs of three biological replicates (*p < 0.05, two-tailed *t*-test).

Growth in SCD_Cd_ medium unbalanced the NAD^+^/NADH ratio (Fig. 3f) and altered the expression profile of genes encoding mitochondrial NADH dehydrogenases which transfer electrons to the respiratory chain. Specifically, *NDI1* (encoding a NADH:ubiquinone oxidoreductase localized on the mitochondrial inner membrane) was upregulated, whereas *NDE1* and *NDE2* (whose products are localized on the mitochondrial external membrane and oxidize cytosolic NADH) were down- and up-regulated, respectively (Fig. 4a)^31^,^32^,^33^.

The significant increase in the mRNA level of *CIT2* (a prototypical retrograde-responsive gene involved in the glyoxylate cycle), *MLS1* and *MDH2* (other glyoxylate cycle genes) observed under calcium shortage (Fig. 4a) suggests an up-regulation of the “retrograde response”, which is usually triggered by mitochondrial dysfunctions and respiration deficiency^34^.

Mitochondrial fragmentation and membrane depolarization have been associated with many yeast death scenarios^35^,^36^. Mitochondrial morphology was examined during growth in SCD and SCD_Cd_ media in cells expressing a mitochondria-targeted variant of green fluorescent protein (mtGFP) and stained with propidium iodide. (Fig. 4b,c; Fig. S8a–b). Essentially, four cell typologies were observed: *(i)* live cells with tubular mitochondria (Tubular/PI^−^, green bars); *(ii)* live cells with a more fragmented, patch-like mitochondrial structure (Fragmented/PI^−^, blue bars); *(iii)* live cells lacking mtGFP signal (GFP^−^/PI^−^, black bars) and *(iv)* dead cells with no mitochondria staining (GFP^−^/PI^+^, red bars). In SCD-log-phase cultures about 90% of the cells belong to the first class, with mitochondria appearing as a branched tubular network distributed at the cell surface (Fig. S8a). In contrast, a significant fraction of live cells with fragmented mitochondria or devoid of mitochondrial signal was detectable in calcium-depleted medium. All dead cells under calcium shortage showed no mitochondrial GFP signal. Following a shift from SCD to SCD_Cd_ medium (Fig. 4e) mitochondria fragmentation was observed as early as 120 minutes after the shift under calcium shortage (blue circles). Live cells lacking any mitochondrial signal (black circles) appeared at about 300 minutes, when dead cells (red circles) were also clearly present.

In summary, these data indicate that mitochondrial fragmentation is an early event following calcium depletion. Both mitochondrial fragmentation and disappearance take place in live cells and can be relieved by forcing respiratory metabolism (*hxk2 hxk1* mutant strain: Fig. S8c).

Nonetheless, the percentage of viable cells competent to respire within the population^37^ (IRC, index of respiratory competence, which reflects the status of mitochondrial functionality) was close to 100% during exponential growth in SCD_Cd_ medium (Fig. 4f) and remained stable during early stationary phase, even though calcium shortage severely reduced chronological lifespan (Fig. S9a–e). These findings indicate that despite the alterations in mitochondrial morphology that accompany the early loss of cell viability during a shift in low-calcium medium, functionality of the organelle is mostly retained in cells that eventually adapt to grow under calcium shortage.

In yeast, electron leakage from the mitochondrial respiratory chain is a major source of ROS^38^. Nevertheless, ROS accumulation in SCD_Cd_ medium did not require a functional respiratory chain: in fact, respiratory-deficient cells (*ρ*^*0*^ *petites* missing mtDNA or *afo1* mutants^39^) showed reduced viability and ROS accumulation just like their isogenic wild-type counterparts (Fig. 4g,h). Experiments with the *yno1* strain (lacking the only known NADPH oxidase in yeast, an ER-resident enzyme that produces superoxide^40^) yielded similar results (Fig. 4g,h).

### Calcium shortage causes sustained Endoplasmic Reticulum (ER) stress and activates the Unfolded Protein Response (UPR)

Since the oxidative stress under calcium shortage did not seem to originate from mitochondria and NADPH oxidase, we tested whether it was generated by protein folding in the ER (accounting for up to 25% of cellular ROS production in yeast^15^). Depletion of calcium ions from the ER by chelators has been shown to induce ER stress and stimulate the UPR signalling network^6^. While transient UPR activation promotes cell survival by reducing protein aggregates and maintaining ER integrity and secretory pathway function, its prolonged activation and failure to relieve persistent ER stress can ultimately result in ROS accumulation and cell death^9^,^10^,^11^,^12^,^13^,^14^,^15^. However, neither oxidative stress nor decreased viability have been observed so far in calcium-starved yeast cell^6^,^17^,^18^,^19^,^41^.

**Figure 5:**
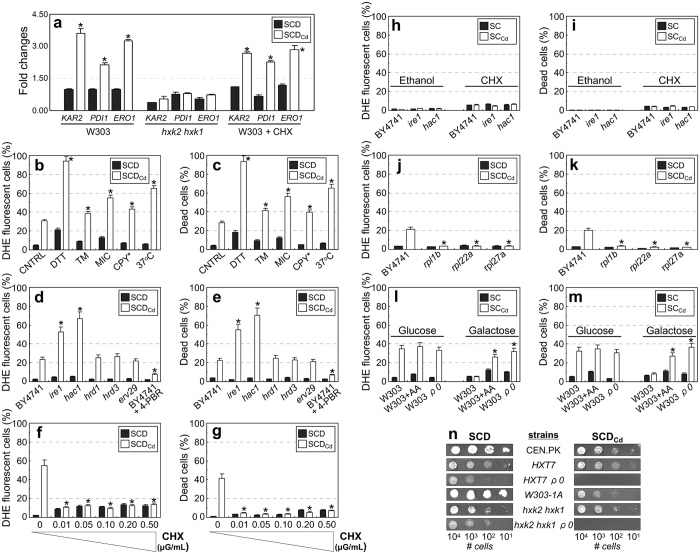
Calcium shortage causes sustained Endoplasmic Reticulum (ER) stress and activates the Unfolded Protein Response (UPR). (**a**) qRT-PCR analysis of gene targets of the Unfolded Protein Response (UPR). Values are means ± SDs of at least biological duplicates (*p < 0.05, two-tailed *t*-test). (**b,c**) SCD-grown cells were transferred either in SCD or SCD_Cd_ media containing the ER stressors dithiothreitol (DTT, 10 mM), tunicamycin (TM, 10 μg/mL) or miconazole (MIC, 10 μM). CPY* cells expressed a misfolded form of the vacuolar carboxypeptidase Y. ROS accumulation and cell viability were evaluated after 24h by cytofluorimetry. Values are means ± SDs of biological triplicates (*p < 0.05, two-tailed *t*-test). (**d,e**) ROS accumulation and cell viability (evaluated by cytofluorimetry) in mutants defective in the UPR response. Where indicated, cells where treated with 10 mM 4-phenylbutyrate (4-PBA). Values are means ± SDs of biological triplicates (*p < 0.05, two-tailed *t*-test). (**f,g**) SCD-cultivated cells were transferred in either SCD or SCD_Cd_ media containing cycloheximide (CHX) at the indicated concentrations. ROS accumulation and cell viability were evaluated by cytofluorimetry 480 min after the shift. Values are means ± SDs of biological triplicates (*p < 0.05, two-tailed *t*-test). (**h,i**) UPR mutants were cultivated either in ethanol medium (respiratory metabolism) or in the presence of 0.01 μg/mL cycloheximide. ROS and cell viability were evaluated by cytofluorimetry. Values are means ± SDs of two biological replicates (*p < 0.05, two-tailed *t*-test). (**j,k**) Slow-growing mutants lacking nonessential ribosomal proteins were shifted in either SCD or SCD_Cd_ media. ROS and cell viability were evaluated after 24h by cytofluorimetry. Values are means ± SDs of two biological replicates (*p < 0.05, two-tailed *t*-test). (**l–n**) Inhibition of respiratory metabolism exacerbates the effects of calcium shortage l–n) wild-type and an isogenic *bona fide ρ*^*0*^ mutant strains were cultivated in SC or SC_Cd_ media supplemented with either glucose or galactose. Where indicated, antimycin A (AA, 1 μg/ml) was added to inhibit cellular respiration. ROS (l) and cell viability (m) were evaluated by cytofluorimetry. Values are means ± SDs of biological triplicates (*p < 0.05, two-tailed *t*-test). (**n**) Cellular suspensions of mutants exhibiting constitutive respiratory metabolism and their isogenic *ρ*^*0*^ counterparts were spotted on either SCD or SCD_Cd_ plates.

The UPR targets Kar2, Pdi1 and Ino1 were significantly up-regulated in cells cultivated under calcium shortage (Fig. 1l; Table SII). These findings were confirmed and expanded by qRT-PCR analysis (Fig. 5a). SCD_Cd_-grown cells were more sensitive to agents or conditions known to induce ER stress, including treatment with the reductant dithiothreitol (DTT), the N-glycosylation inhibitor tunicamycin (TM) and the antifungal drug miconazole (MIC, which targets ergosterol biosynthesis in the ER), as well as by overexpression of a misfolded vacuolar carboxypeptidase Y (CPY*) and incubation at 37 °C (Fig. 5b,c). Additionally, the phenotypes associated to calcium shortage were strongly exacerbated in mutants defective in UPR signaling (*ire1* and *hac1*), which exhibited marked ROS accumulation and viability loss in our experimental set-up (Fig. 5d,e).

While inability to remove misfolded proteins by either the ER-associated degradation (ERAD, involving *HRD1* and *HRD3*) or the HIP-dependent pathway (involving *ERV29*)^42^ did not affect the penetrance of calcium starvation phenotypes, 4-phenylbutyrate treatment (4-PBA, a chemical chaperone which increases protein folding capacity^43^) significantly mitigated the effects of calcium shortage (Fig. 5d,e).

Calcium shortage-induced ROS accumulation and cell death were substantially abolished by pre-treatment with sub-lethal doses of cycloheximide (an inhibitor of protein translation) and in slow-growing mutants exhibiting reduced translation rate due to the loss of nonessential ribosomal proteins (Fig. 5f–i; Fig. S10a–c). These results support the notion that the enhanced ROS production and cell death induced by calcium shortage are mediated by ER stress and may be relieved by decreasing the protein load in the ER lumen through a reduction in the overall translation rate^44^.

## Discussion

**Figure 6:**
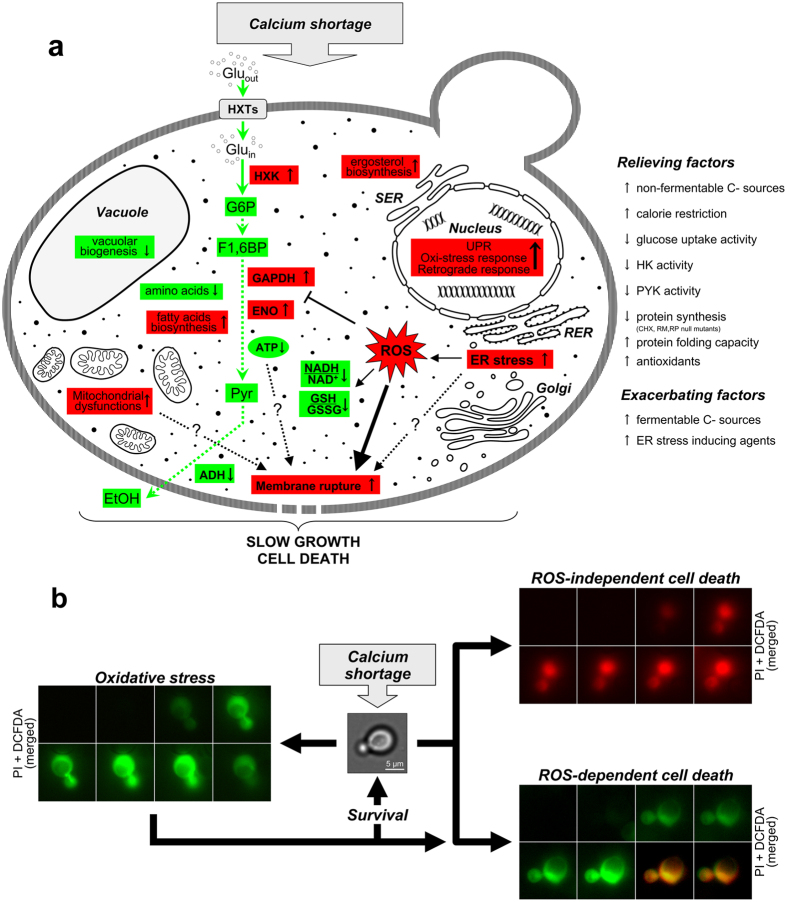
Effects of calcium shortage on yeast physiology and their modulation by genetic and environmental factors. (**a**) Red indicates upregulation, green downregulation. RER: Rough Endoplasmic Reticulum; SER: Smooth Endoplasmic Reticulum; ROS: reactive oxygen species; HXTs: hexose transporters; HK: hexokinase; GAPDH: Glyceraldehyde-3-phosphate dehydrogenase; ENO: enolase; ADH: alcohol dehydorgenase; G6P: glucose 6-Phosphate; F1,6BP: Fructose 1,6-bisphosphate; Pyr: pyruvate; EtOH: ethanol. See text for details. (**b**) Possible fates of calcium-starved yeast cells. Cells were stained with DCFDA before the shift in calcium-depleted medium containing PI. Shown are representative dual-channel merged fluorescence time-lapse series (acquired at 15 min intervals) depicting all the possible outcomes for a calcium-starved yeast cell.

Tight regulation of calcium-triggered signaling mechanisms is crucial to the survival of all life forms. Previous works provided conflicting evidence about the effects of calcium shortage on cell physiology in the model eukaryote *Saccharomyces cerevisiae*^6^,^17^,^18^,^19^. Here we show that a general slowdown of cell growth, enhanced ROS production and a decrease in cell viability are the most distinctive hallmarks of calcium-starved yeast cells (Fig. 6).

The oxidative stress in yeast cells grown under calcium shortage may result from accumulation of misfolded proteins within the ER lumen (ER stress), which has been associated with ROS production and cell death^6^,^9^,^10^,^11^,^12^,^13^,^14^,^15^,^41^. Consistently, many UPR targets are significantly up-regulated in calcium-starved cells and mutants defective in UPR signaling exhibit enhanced sensitivity to calcium deprivation (Fig. 5a–e).

Furthermore, ROS accumulation and calcium shortage-induced cell death are exacerbated by ER-stressing conditions and relieved by treatment with the 4-PBA chemical chaperone, which stabilizes protein conformation and improves ER folding capacity, confirming that the ER function is severely compromised in calcium-starved cells (Fig. 5b–e). Enhanced glycerol biosynthesis (an osmolite behaving as chemical chaperone^45^) occurring under calcium shortage (Fig. 2b; Table SII) may be part of the protective mechanisms against ER stress.

ER homeostasis, UPR signaling and lipid metabolism are tightly linked in both yeast and metazoans^46^,^47^. The highly oxidizing intracellular environment occurring under calcium shortage likely damages cellular components, including lipids: the increased production of palmitate and oleate and the up-regulation of several ER-localized enzymes involved in ergosterol biosynthesis (the major yeast sterol)^48^ may contribute to regenerate damaged membranes and to provide building blocks for ER expansion, required to cope with increased demand for protein folding capacity^49^ (Fig. 2b; Table 2; Table SII). Alternatively, disturbance of lipid homeostasis by hyperaccumulation of fatty acids and sterols may also impair ER function and activate UPR, as reported for both yeast and human cells^47^,^50^,^51^,^52^.

Calcium signaling *via* calcineurin is essential for survival of yeast cells exposed to inhibitors of essential ER processes^6^,^9^,^12^ and several death scenarios have been associated with failure to handle prolonged ER stress^15^. Under calcium shortage, ROS accumulation anticipates cell death (Fig. 3s) in an active process that requires *de novo* protein synthesis (Fig. 5f,g; Fig. S10a–c) and Kex1, a protease involved in other yeast death scenarios, including the demise of cells exposed to tunicamycin^13^,^29^. Cell death is not dependent on apoptotic effectors and is not associated with the morphological hallmarks of apoptosis (Fig. 1e,f), thus suggesting a non-apoptotic program, whose precise nature remains to be elucidated.

The partial effect of calcium shortage on yeast viability is in keeping with recent reports that cell heterogeneity can originate purely at the metabolic level^53^, a property of complex metabolic and regulatory systems that plays a relevant role in multifactorial diseases like cancer. Indeed, not all calcium-starved cells follow the pathway to death. Some ROS-producing cells adapt and remain viable under calcium shortage, presumably by strong up-regulation of the oxidative stress response (Fig. 6b): since at steady state viable cells account for about 70% of the whole population, this pathway is likely the prevailing one. Both a pre-activation of the oxidative-stress response and anti-oxidant treatments can significantly increase the survival of calcium-depleted cells (Fig. 3r; Fig. S7h), indicating that the predominant death pathway is ROS-dependent. Time-lapse experiments confirmed that ROS-positive, calcium-starved cells may eventually lose membrane integrity and die (Fig. 6b). Additionally, a second, apparently ROS-independent death route exists under calcium shortage, as suggested by the presence of ROS-negative dead cells (Fig. 3s; Fig. 6b; Fig. S7). The low, steady state concentration of these cells suggests that this is a minor route. Nonetheless, the subpopulation of ROS-negative dead cells might be underestimated, if either non-specific staining of dead cells with ROS probes or *post mortem* ROS production occurs.

The effects of calcium shortage are strictly carbon source-dependent (and thus, ultimately, metabolism-dependent). In media supplemented with rapidly fermentable sugars, yeast cells typically grow fast, obtaining energy mainly through fermentation^23^,^54^,^55^. While growth on glucose allows to sustain strong protein influx into the ER, calcium shortage compromises the ER function, slow down the proliferation rate and reduces the glycolytic flux (Fig. 2b; Table 1; Fig. 6; Table SI) with no concurrent activation of respiration (as shown by the unchanged glucose-to-ethanol ratio and by the transcriptional profile: Fig. 2a; Fig. S4a). The lower intracellular ATP level (Table 1) observed in SCD_Cd_-grown cells may result from the combined reduced production and the extra consumption needed to sustain increased lipid synthesis.

Cells cultivated on poorly/non-fermentable carbon sources or under calorie restriction regimen adopt a respiratory metabolism, grow slowly and are insensitive to calcium shortage (Fig. 2c–f; Table 1; 5h,i; Fig. S6a; Table SI). Genetic modulations of metabolism that favor respiration and slow growth (*hxk, hxt* and *pyk* mutants^24^,^26^,^27^) also rescue the defects induced by calcium shortage (Fig. 2g; Fig. S6b; Fig. S8c). Each of these situations *indirectly* decreases protein translation, since respiratory metabolism is accompanied by reductions in macromolecular syntheses and growth rate^56^,^57^: therefore, in slow-growing, respiring cells the protein influx into the ER may remain within the capacity of the folding machinery even in low-calcium medium, thus preventing ER stress and the insurgence of the harmful effects of calcium deprivation. Accordingly, the effects of calcium shortage are also mitigated by conditions that *directly* decrease the overall protein synthesis rate by chemical (cycloheximide pretreatment: Fig. 5f–i; Fig. S10a–c) or genetic means (inactivation of genes encoding nonessential ribosomal subunits: Fig. 5j–k; Fig. S8c)^44^. Strikingly, the high sensitivity of UPR mutants to calcium shortage was suppressed both by cycloheximide treatment or by forcing respiratory metabolism (Fig. 5h,i), although only respiratory metabolism could abolish the UPR activation in low calcium medium (Fig. 5a). This is not completely surprising, since reduction of the overall protein synthesis rate is just one of the mechanism by which UPR activation counteracts ER stress.

Conversely, the effects of calcium shortage are exacerbated when yeast cells are forced to adopt a purely fermentative (albeit inefficient) metabolism. Wild-type cells treated with antimycin A (an inhibitor of cellular respiration) and their isogenic *ρ*^*0*^ counterparts exhibit ROS accumulation and decreased viability during growth in SC_Cd_ medium supplemented with galactose, a poorly fermentable carbon source (Fig. 5l,m). Respiratory-deficient mutants with an intrinsically inefficient fermentative metabolism (*hxk1 hxk2 ρ*^*0*^ and *HXT7 ρ*^*0*^ strains) grow at extremely reduced rate under optimal conditions but are unviable under calcium shortage (Fig. 5n), indicating that slow growth rescues calcium shortage-related phenotypes only if coupled to respiration (and ensuing ATP production).

In mammalian cells prolonged depletion of intraluminar ER calcium (a condition associated with many human diseases) affects the activity of ER-resident chaperones and is a potent inducer of ER stress that triggers the UPR signaling and can lead to apoptosis^58^,^59^. Our findings confirm that the connections among ER calcium homeostasis, ER stress and UPR signaling originally described in human cells^58^,^59^ are evolutionary conserved in yeast, as previously suggested^6^ but never fully explored. For the first time we show that the phenotypes induced by calcium shortage in yeast can be rescued by decreasing the rate of protein synthesis and/or by forcing cellular respiration, indicating that the fate of calcium-starved cells is dictated by their ability to balance energy demands for protein synthesis and the mode of energy production (i.e. fermentative *vs* respiratory).

Our work may contribute to improve the understanding of several human pathologies (including diabetes, cardiovascular dysfunctions, viral infections, cancer and neurodegenerative disorders) in which ER calcium deficiency has been recognized a role^58^.

## Methods

### Strains, plasmids and growth conditions

*Saccharomyces cerevisiae* strains and plasmids used in this study are listed in Table 3. Recombinant DNA manipulation and yeast transformation were performed according to standard protocols. To allow growth on maltose, the wild-type strain W303-1A was transformed with two plasmids containing respectively the *MALS* and *MALT* genes cloned under the strong constitutive *TPI* promoter into the integrative plasmids pYX012 (*TPI*_*pr*_-*, URA3)* and pYX042 (*TPI*_*pr*_-*, LEU2)*^60^.

B*ona fide* W303-1A *ρ0* strains lacking mitochondrial DNA were generated according to the classical ethidium bromide procedure^61^.

The strain W-GUS (W303-1A *HSP12::GUS*) used for the *β-*glucoronidase assay was obtained by transforming W303-1A with the integrative plasmid pKV3-d2 containing the *HSP12* promoter region fused to the *GUS* reporter gene^62^.

Deletion strains in the BY4741 background were obtained from the EUROSCARF collection (www.euroscarf.de).

The entire set of strains with reduced pyruvate kinase activity^26^ was kindly provided by Prof. Markus Ralser (University of Cambridge, UK).

The *hxt*-null strain EBY.VW4000^63^ was a kind gift from Prof. Eckhard Boles (Goethe-Universitaet Frankfurt, Germany).

The integrative plasmids pYX022-*HXT1* and pYX022-*HXT7 (TPI*_*pr*_-*, HIS3)*^64^ for the constituitive expression of *HXT* genes were a kind gift from Prof. Paola Branduardi (University of Milano-Bicocca, Italy).

Plasmid pPW2427 (*GPD*_*pr*_-CPY*-HA, *CEN, URA3*)^65^ was kindly provided by Prof. Peter Walter (University of California, USA).

pVTU-mtGFP (*ADH1*_*pr*_-mtGFP, *2μ, URA3*)^66^ was obtained by the Addgene repository (Addgene plasmid 45054).

Plasmid YEplac195*-HXK2 (2μ, URA3)*^67^ was a gift from Prof Johan Thevelein (KU Leuven, Belgium).

Cultures were grown in synthetic complete (SC) medium, containing 0.67% (w/v) yeast nitrogen base (YNB) without amino acids and appropriate quantities of the “drop-out” amino acid-nucleotide mixture (Complete Synthetic Mixture, Q-Biogene). SC medium contains 680.2 μm CaCl_2_ and 0.8 μm calcium pantothenate. Calcium depleted media (SC_Cd_) were prepared using 0.66% (w/v) YNB-Ca^2+^ (Q-Biogene), in which CaCl_2_ was omitted and calcium pantothenate replaced with sodium panthotenate: residual calcium concentration in these media has been reported to be at least 2500 fold lower than standard YNB^17^. MilliQ water was used for media preparation in order to avoid Ca^2+^ contamination. The carbon source was added at 2% (w/v) final concentration, unless otherwise stated. Cells were grown in flasks at 30 °C on a rotary shaker. Growth of cultures was monitored as increase in cell number using a Coulter Counter model Z2 (Coulter Electronics, Inc.). The fraction of budded cells was scored by direct microscopic observation on at least 300 cells, fixed in 3.6% (v/v) formaldehyde and mildly sonicated.

### Determination of DNA, RNA and protein contents by flow and cytometry and chemical dosage

For cytofluorimetric determination of DNA, RNA and protein intracellular content, samples were essentially processed as previously described^68^,^69^.

### Elutriations and flow cytofluorimetric analysis

Centrifugal elutriation was performed from 3 liters cultures grown to mid-exponential phase, essentially as previously described^70^. Cells were separated according to their sizes using a 40 ml chamber elutriator (Beckman Coulter).

### Viability assays and tests for apoptotic/necrotic markers

The fraction of dead cells within the population was quantified by flow cytometry after staining with 6 μg/ml propidium iodide and confirmed by direct microscopic examination of at least 500 cells stained with trypan blue or methylene blue. In order to determine chronological life span (CLS), cell viability was monitored over time by both flow cytometry and by clonogenic survival plating assays^37^. Exposed phosphatidylserine and loss of plasma membrane integrity were detected by Annexin-V/propidium iodide (AnnV/PI) co-staining. After staining, spheroplasts were observed under fluorescence microscope and analyzed quantitatively by flow cytometry^71^.

### Vacuole staining

For vacuole visualization, cells were stained with 80 μM FM4-64 (N-(3-triethylammoniumpropyl)-4-(p-diethylaminophenylhexatrienyl) pyridinium dibromide, Molecular Probes, Invitrogen) or with 500 μM CDC-FDA (5-(and-6)-carboxy-2’,7’-dichlorofluorescein diacetate, Molecular Probes, Invitrogen), essentially as described^72^. Dual staining for simultaneous visualization of vacuolar morphology and cell death was performed with 80 μM FM4-64 and 50 μM Sytox green.

### Study of mitochondrial morphology and functionality

Mitochondrial morphology was examined in cell constitutively expressing a mitochondria-targeted GFP variant (mtGFP)^66^, stained with 6 μg/ml propidium iodide. The classification of the mitochondrial network morphology as tubular or fragmented in viable and dead cells was performed by direct observation of at least 500 cells under a Nikon Eclipse E600 fluorescence microscope, equipped with a 100X, 1.4 oil Plan-Apochromat objective and standard fluorescein filter set. Images were digitally acquired using a Leica DC 350F camera and processed with ImageJ (http://imagej.nih.gov/ij/).

The index of respiratory competence (IRC) was determined by spreading identical amounts of yeast cells on YP medium plates supplemented with either fermentable (YPD, glucose) or nonfementable (YPEG, Ethanol/Glycerol) carbon sources. The IRC value was calculated as the ratio between the number of colony-forming units (CFUs) observed on YPEG vs YPD plates^37^.

### ROS accumulation

Free intracellular radicals accumulation was detected by staining samples with either 10 μg/ml dichlorodihydrofluorescein diacetate (DCFDA, oxidized by ROS to fluorescent dichlorofluorescein) or 5 μg/ml dihydroethidium (DHE, specifically oxidized by superoxide ions to fluorescent ethidium), essentially as described^73^. Dual staining for simultaneous detection of ROS accumulation and cell death was performed with either 10 μg/ml DCFDA plus 6 μg/ml PI or 5 μg/ml dihydroethidium plus 50 μM Sytox Green (Thermofisher). At least 500 cells were scored by direct observation under a fluorescence microscope. Alternatively, intracellular ROS levels were quantified by flow cytometric analysis using a FACScan instrument (Becton Dickinson).

### Time-lapse microscopy

Approximately 5*10^5^ mildly sonicated SCD-grown cells were immobilized on the glass surface of a 35 mm glass-bottom dish coated with Concanavalin A (100 μg/m; ~5000 cells per 1 mm^2^ glass surface). Immobilized cells were stained for 1 hour with CDCFDA (10 μg/ml in 3 mL of SCD medium), washed twice with fresh SCD_Cd_ medium and covered with 3 mL of the same medium containing 6 μg/ml PI.

Time-lapse experiments were performed by using a Nikon A1R inverted microscope equipped with a 100× oil immersion objective (NA 1) and a Andor camera (NEO 5.5 sCMOS). Temperature (30 °C) and humidity were controlled throughout the measurement by a Okolab incubating system. DCFDA (535 nm) and PI (620 nm) emission signals were acquired every 15 minutes for about 10 hours (exposure time 200 ms). Focusing was maintained throughout the measurement by Nikon perfect focus system. At longer incubation times cells tend to detach from the plate floating out of the field. Given that quantitative single cell analysis was limited by cell motion and duplication during the time lapse experiment, a population analysis was performed by using the NIS Elements imaging software (v. 4.5). Mean CDCFDA and PI fluorescence was extrapolated from each image after background subtraction and plotted vs time. Fiji software (http://fiji.sc) was used for image post-processing.

### Enzymatic assays

The hexokinase assay was performed essentially as described^74^, using a glucose-oxidase-peroxidase reaction kit (Sigma-Aldrich). GAPDH activity assay was performed essentially as described^75^, measuring the rate of NADH formation per minute, following the increase in absorbance at 340 nm. The assay for invertase activity was performed essentially as previously described^68^. The *β-*glucoronidase enzymatic assay was performed as described^76^. Glucose consumption and ethanol production were evaluated by standard enzymatic assays (Sigma-Aldrich; Megazyme), essentially as previously described^68^.

### Quantification of intracellular NADH/NAD^+^

Yeast cells cultures in middle exponential phase were quenched as described^77^. Harvested cells were resuspended in 0.3 ml of 0.2M NaOH for NADH determination or 0.2M HCl for NAD^+^ determination. Cell suspensions were incubated at 55 °C for 10 minutes and clarified from cell debris. The cycling assay for and NADH NAD^+^ determination was performed according to^78^ by monitoring the reactions at 575 nm over time.

### Evaluation of stress resistance

Aliquots of exponentially growing cells were either heated at 51 °C (heat-shock) or treated with hydrogen peroxide (oxidative stress), lithium chloride (ionic stress) or sorbitol (osmotic stress) as indicated in Fig. S5. Treated and untreated cells were then serially diluted, plated on YPD plates and incubated for 2 days at 30 °C to obtain viable counts (CFUs). Endoplasmic Reticulum (ER) Stress was induced by treating cells with either the reducing agent dithiothreitol (DTT), or the N-glycosylation inhibitor tunicamycin (TM), or the antifungal miconazole (MIC). In addition, the effects of constitutive overepression of CPY* (a misfolded variant of vacuolar carpoxypeptidase Y^65^) were evaluated during growth at both optimal (30 °C) and elevated (37 °C) temperatures. In order to limit accumulation of misfolded proteins inside the ER lumen, cells were cultivated in the presence of either the chemical chaperone sodium phenylbutyrate (4-PBR, 10 mM) or sublethal doses of the protein synthesys inhibitor cyclohehimide (CHX, 0.05–0.50 μg/mL).

### Protein extraction, 2D-PAGE and gel image analysis

Protein extraction, 2DE, *in situ* digestion of gel spots, peptide extraction and MALDI-MS analysis were performed as previously described^72^. Proteins showing a reproducible fold change ≥1.5 upon SCD *vs* SCD_Cd_ comparison were considered consistently changing. The online tool Genecodis (http://genecodis.cnb.csic.es/) was used for the functional class analysis of the proteins which showed a differential expression between SCD and SCD_Cd_ conditions^79^. The resulting list of enriched Gene Ontology Biological Process terms were filtered by REVIGO (http://revigo.irb.hr/)^22^ in order to remove redundant terms and the results were visualized as treemaps in which related terms are joined into loosely related “superclusters”. Size of the tiles reflects the *p*-value.

### Intracellular metabolite extraction and analysis

Yeast cells cultures in middle exponential phase were quenched according to^77^. Metabolite extraction was performed using the pure methanol method^80^. Amino and non-amino organic acid levels were determined by GC-MS analysis as described^80^. Peaks were identified using an in-house MS library and data analysis was performed using AMDIS (Automated Mass Deconvolution and Identification System) and R^81^. Values were normalized by internal standard (i.e. chlorophenylalanine) and by cell dry weight of each sample. Extracellular metabolites were analysed as described^82^. For the determination of intracellular adenine nucleotides and glycolytic intermediates, metabolites were extracted according to the boiling buffered ethanol procedure^83^. ATP was quantified by a luciferin-luciferase assay using the ATPlite™ kit (Perkin Elmer) according to manufacturer’s instructions. ADP and AMP were measured after enzymatic conversion to ATP by pyruvate kinase and myokinase, essentially as previously described^84^. Glucose-6-phosphate was quantified with an enzymatic fluorimetric assay as reported^85^. For the detection of fructose-6P and glucose-1P, phosphoglucoisomerase (1 U/ml) and phosphoglucomutase (1U/mL) were added to the reaction mix, respectively. Fructose 1,5 bisphosphate, Dihydroxyacetone phosphate, Glyceraldehyde 3-phosphate, phosphoenolpyruvate and pyruvate intracellular content was determined by appropriate enzimatic methods, as described^86^. Quantification of glutathione levels was performed as previously described^68^,^87^. For each metabolite, the intracellular concentration was estimated by dividing the measured values by the mean cellular volumes (as evaluated by Coulter counter analysis).

### Quantitative Real-time PCR

Total RNA was prepared as described previously^68^. qRT-PCR reactions were performed in a MiniOpticon detection system (BIO-RAD) using the SsoFast EvaGreen Supermix (BIO-RAD). Primer sequences are available on request. Data obtained were analyzed with the CFX Manager software (BIO-RAD) and normalized to the transcript levels of the *TAF1* and *CDC34* housekeeping genes within the same sample.

### Statistical analysis

Data are reported as means ± SDs from at least three independent experiments. Statistical significance (indicated with “*”) of the measured differences was assessed by two-sided Student’s t-test (p < 0.05).

## Additional Information

**How to cite this article**: Busti, S. *et al*. Respiratory metabolism and calorie restriction relieve persistent endoplasmic reticulum stress induced by calcium shortage in yeast. *Sci. Rep.* **6**, 27942; doi: 10.1038/srep27942 (2016).

## Supplementary Material

Supplementary Information

Supplementary Information

## Figures and Tables

**Table 1:** Glycolytic metabolites and adenylate energy charge in cells grown in SC and SC_Cd_ media.

	Metabolite concentration (mM)					
	*2% Glucose*		*0.05% Glucose*+		*2%EtOH*	
	*SC*	*SC*_*Cd*_	*SC*	*SC*_*Cd*_	*SC*	*SC*_*Cd*_
*Glucose-6P*	2.72 ± 0.40	0.46 ± 0.10	0.24 ± 0.02	0.20 ± 0.03	0.16 ± 0.01	0.19 ± 0.02
*Fructose-6P*	0.15 ± 0.04	0.06 ± 0.01	0.04 ± 0.01	0.03 ± 0.01	0.02 ± 0.01	0.03 ± 0.01
*Fructose-1,6BP*	2.88 ± 0.20	0.48 ± 0.07	0.34 ± 0.03	0.30 ± 0.03	0.74 ± 0.07	0.55 ± 0.11
*Pyruvate*	0.77 ± 0.11	0.51 ± 0.07	0.35 ± 0.10	0.29 ± 0.08	0.13 ± 0.01	0.12 ± 0.02
*ATP*	2.61 ± 0.09	1.34 ± 0.06	3.44 ± 0.18	3.09 ± 0.21	3.33 ± 0.20	2.97 ± 0.15
*ADP*	0.89 ± 0.15	0.46 ± 0.10	0.95 ± 0.11	0.87 ± 0.12	0.98 ± 0.18	1.03 ± 0.13
*AMP*	0.21 ± 0.04	0.08 ± 0.03	0.24 ± 0.07	0.22 ± 0.07	0.32 ± 0.08	0.26 ± 0.06
Energy Charge	0.82	0.84	0.85	0.84	0.83	0.82
*GSH*_*Tot*_	3.82 ± 0.35	5.24 ± 0.79	4.70 ± 0.56	8.39 ± 0.84	ND	ND
*GSH/GSSG*	46.5 ± 5.2	24.9 ± 4.4	54.4 ± 4.8	45.6 ± 3.1	ND	ND

Metabolites were extracted from cells grown till mid exponential phase (~2*10^7 ^cells/mL, ~1*10^7 ^for 0.05% glucose coltures) in SC or SC_Cd_ supplemented with the indicated carbon sources and assayed by enzymatic analyses according to the procedures described in Materials and Methods.

For each metabolite, the intracellular concentration was estimated by dividing the measured values by the mean cellular volumes (as evaluated by Coulter counter analysis). Values reported are means ±SDs of at least two independent experiments.

The adenylate energy charge was calculated according to the formula

([ATP] + 1/2 [ADP])

([ATP] + [ADP] + [AMP]).

**Table 2:** Variation of identified intracellular metabolites between growth in SC and SC_Cd_ media.

Metabolite	fold change SC_Cd_/SC	
	2% glucose	0.05% glucose
Valine	+4.3	–
Oleic acid	+3.6	–
Citric acid	+3.0	–
Stearic acid	+2.1	–
Palmitic acid	+0.7	–
Alanine	–	+6.0
Leucine	−1.9	–
Norleucine	−3.0	–
Methionine	−2.9	–
Glycine	−4.2	–
Glutamic acid	−4.9	–
Serine	−6.0	–
Lysine	7.4	–
Proline	−8.0	–
Aspartic acid	−20.6	–

The analysis was performed via GC-MS allowing the identification of 55 total metabolites (mainly organic acids). Fold change values are reported only for metabolites with statistically significant variation (p-value ≤ 0.01).

**Table 3:** Strains used in this study.

Strains	Relevant genotype	Reference
W303-1A	MATa leu2-3,112 ura3-1 trp1-1 his3-11,15 ade2-1 can1-100 GAL SUC mal	88
W303-1A ρ°	W303-1A [ρ°]	This study
YSH599 (hxk2)	W303-1A hxk2::LEU2	89
W-GUS	W303-1A HSP12::GUS::URA)	This study
YSH601 (hxk2 hxk1)	W303-1A hxk1::HIS3 hxk2::LEU2	89
hxk1 hxk2 ρ°	W303-1A hxk1::HIS3 hxk2::LEU2 [ρ°]	This study
HXK2	W303-1A [YE-HXk2]	This study
snf1	W303-1A snf1::kanMX	This study
SP1	MATa his3 leu2 ura3 trp1 ade8 Can	90
CEN.PK2-1C	MATa ura3-52 trp1-289 leu2-3,112 his3Δ 1 MAL2-8C SUC2	91
CEN.PK2-1C ρ°	MATa ura3-52 trp1-289 leu2-3,112 his3Δ 1 MAL2-8C SUC2 [ρ°]	This study
EBY.VW4000 (hxt(1-7) gal2)	CEN.PK2-1C hxt13 ::loxP hxt15::loxP hxt16::loxP hxt14 ::loxP hxt12::loxP hxt9::loxP hxt11::loxP hxt10::loxP hxt8::loxP hxt514::loxP hxt2::loxP hxt367 ::loxP gal2 stl1::loxP agt1::loxP ydl247w::loxP yjr160c::loxP	63
hxt(1-7) gal2 HXT1	EBY.VW4000 TPI_pr_-HXT1::HIS3	This study
hxt(1-7) gal2 HXT7	EBY.VW4000 TPI_pr_-HXT7::HIS3	This study
hxt(1-7) gal2 HXT7 ρ°	EBY.VW4000 TPI_pr_-HXT7::HIS3 ρ°	This study
BY4741	MATa his3Δ 1 leu2Δ 0 met15Δ 0 ura3Δ 0	92
zwf1	BY4741 zwf1::kanMX	EUROSCARF
afo1	BY4741 afo1::kanMX	EUROSCARF
yno1	BY4741 yno1::kanMX	EUROSCARF
ire1	BY4741 ire1::kanMX	EUROSCARF
hac1	BY4741 hrd1::kanMX	EUROSCARF
hrd1	BY4741 hrd3::kanMX	EUROSCARF
hrd3	BY4741 hac1::kanMX	EUROSCARF
erv29	BY4741 erv29::kanMX	EUROSCARF
rpl1b	BY4741 rpl1b::kanMX	EUROSCARF
rpl22a	BY4741 rpl22A::kanMX	EUROSCARF
rpl27a	BY4741 rpl27A::kanMX	EUROSCARF
TEFpr-PYK1	BY4741 pyk1::natMX4 pyk2::kanMX [p413TEF_pr_-PYK1]	26
TEFpr-PYK2	BY4741 pyk1::natMX4 pyk2::kanMX [p413TEF_pr_-PYK2]	26
CYCpr-PYK1	BY4741 pyk1::natMX4 pyk2::kanMX [p413CYC_pr_-PYK2]	26
CYCpr-PYK2	BY4741 pyk1::natMX4 pyk2::kanMX [p413CYC_pr_-PYK2]	26
yca1	BY4741 yca1::kanMX	EUROSCARF
aif1	BY4741 aif1::kanMX	EUROSCARF
nuc1	BY4741 nuc1::kanMX	EUROSCARF
nma111	BY4741 nma111::kanMX	EUROSCARF
kex1	BY4741 kex1::kanMX	EUROSCARF
bxi1	BY4741 bxi1::kanMX	EUROSCARF
slt2	BY4741 slt2::kanMX	EUROSCARF
vma1	BY4741 vma1::kanMX	EUROSCARF
pex6	BY4741 pex6::kanMX	EUROSCARF
pep4	BY4741 pep4::kanMX	EUROSCARF
pep3	BY4741 pep4::kanMX	EUROSCARF
sod1	BY4741 sod1::kanMX	EUROSCARF
sod2	BY4741 sod2::kanMX	EUROSCARF
ctt1	BY4741 ctt1::kanMX	EUROSCARF
cta1	BY4741 cta1::kanMX	EUROSCARF
