# Supplementary material for: Respiratory metabolism and calorie restriction relieve persistent endoplasmic reticulum stress induced by calcium shortage in yeast
Source: Sci Rep. 2016 Jun 16;6:27942. doi: 10.1038/srep27942 (PMC4910072; doi:10.1038/srep27942)
Supplement: Supplementary Information [file srep27942-s1.pdf]

Supplementary Information

Busti S., Mapelli V., Tripodi F., Sanvito R., Magni F., Coccetti P., Rocchetti M., Nielsen J., Alberghina L., Vanoni M.

Respiratory metabolism and calorie restriction relieve persistent endoplasmic reticulum stress induced by calcium shortage in yeast

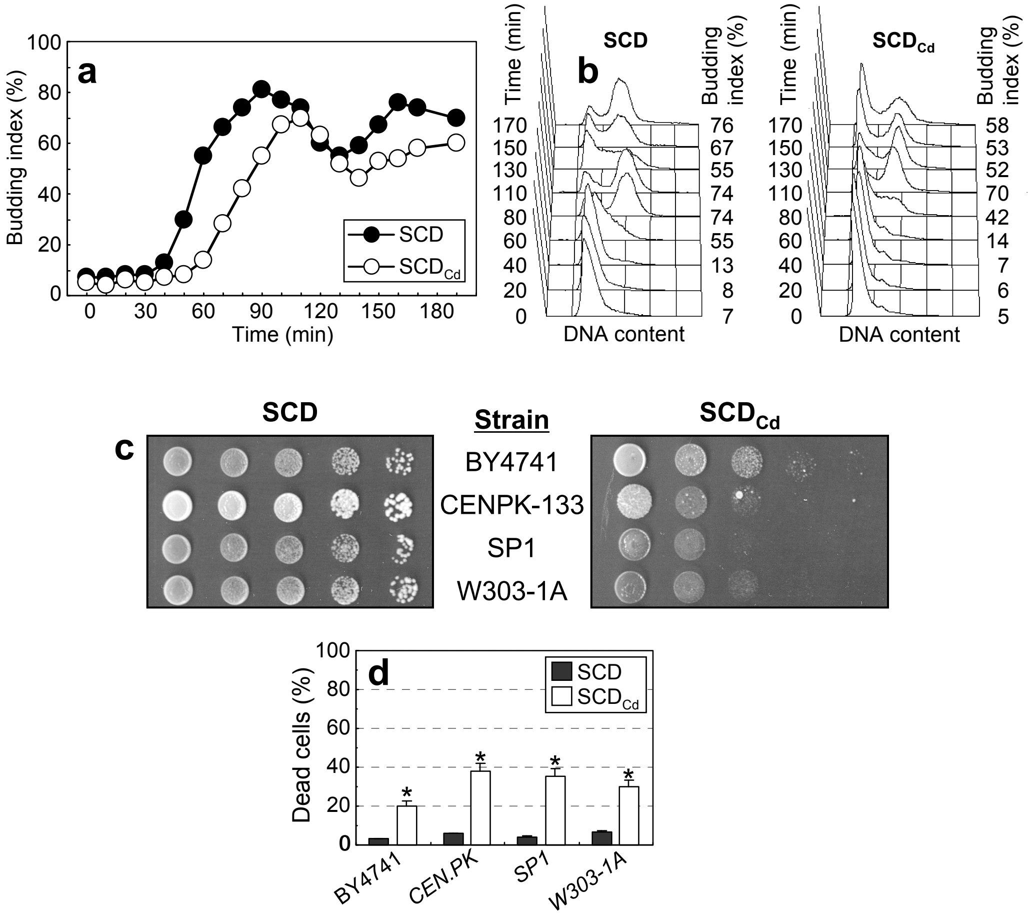

Figure S1 Calcium shortage affects cell growth and viability irrespective of the genetic background

a-b) Calcium shortage causes a general slowdown of cell cycle progression. Cells in G1 phase were sorted by centrifugal elutriation and released at t_0 into SCD or SCD_{Cd} fresh media supplemented with 2% glucose. Growth parameters were monitored for an entire round of cell division cycle. The budding index (panel a) and the DNA distribution profiles (obtained by FACS analysis, panel b) after the release of synchronous cultures in fresh media are shown. In panel b, points through cell cycle are indicated on the left side, whereas the percentage of budded cells referring to each time point is indicated on the right side.

c) Cellular suspensions of several wild type strains were serially diluted and spotted on SC and SC_{Cd} plates supplemented with 2% glucose. Plates were photographed after 48h at 30 ° C.

d) The fraction of dead cells in logarithmic phase cultures cultivated in SC and SC_{Cd} glucose medium was evaluated by clonogenic assay. A direct count of cells stained with either methylene blue or trypan blue yielded similar results. Bars are means ± SDs from two independent experiments (*$p<0.05$, two-tailed t-test).

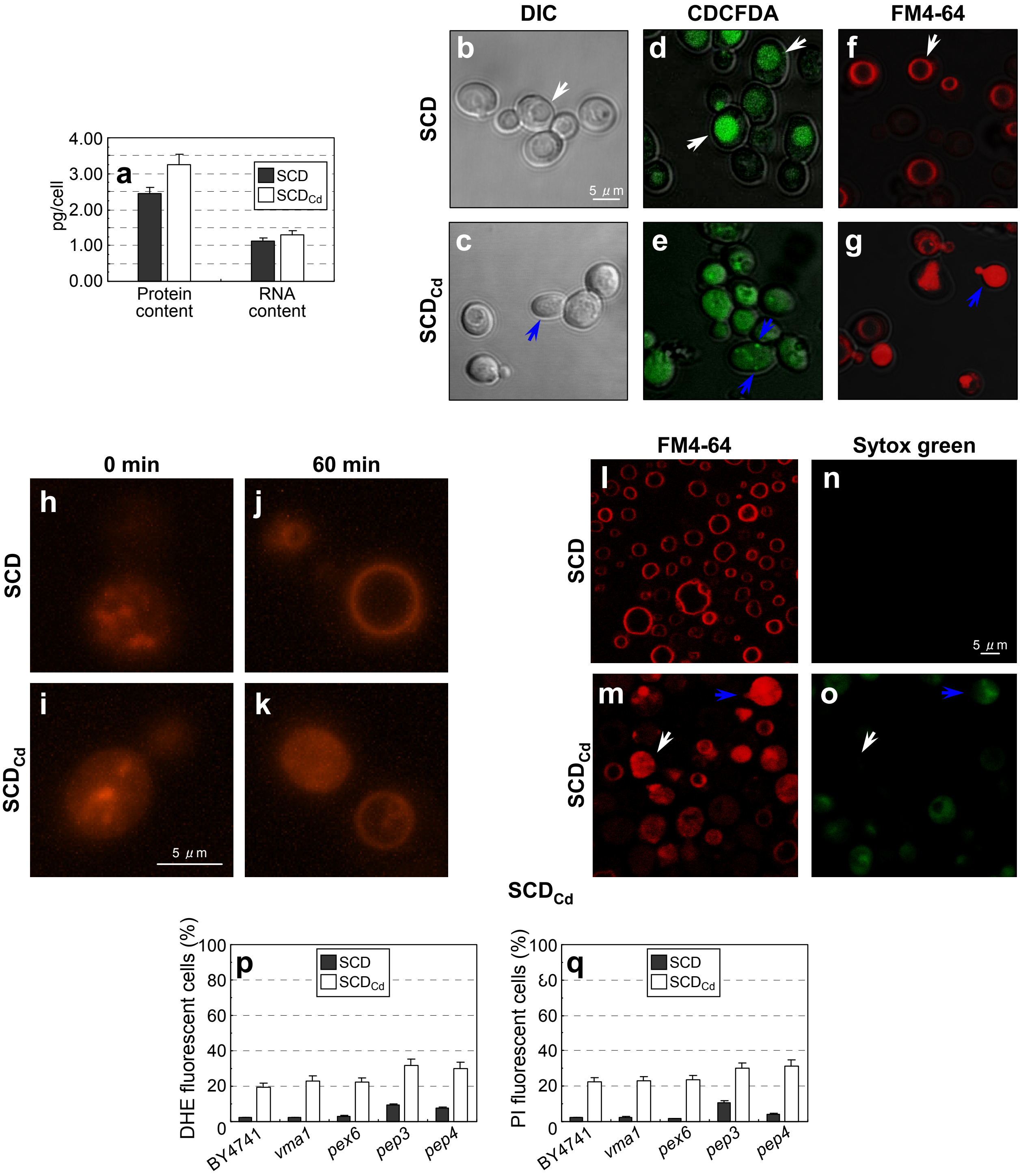

Figure S2 Calcium shortage affects vacuolar morphology

a) Protein and RNA cellular content in cells cultivated in either SCD or SCD_{Cd} glucose medium. Mean values +SDs from two independent measures as determined by chemical dosage are shown.

b-g) Vacuole morphology in cells exponentially growing in either SCD or SCD_{Cd} medium was analysed after staining with either CDCFDA ((5(6)-Carboxy-2',7'-dichlorofluorescein diacetate, a dye for the vacuole lumen (d and e)) or FM4-64 (a dye for the vacuolar cembrane (f and g)). Cells were observed under a microscope with DIC optics (b-c) or appropriate fluorescence filters. White arrows indicate cells with normal vacuoles, blue arrows indicate cells with abnormal vacuolar morphology.

h-k) Kinetic experiments of FM4-64 internalization in cells exponentially growing in SCD or SCD_{Cd} media. Representative fluorescence microscopy images at t = 0 min and t = 60 min are shown.

l-o) FM4-64 and Sytox green double staining of cells exponentially growing in SCD or SCD_{Cd} media. Representative fluorescence microscopy images are shown. The blue arrow indicates a representative dead cell (positive to Sytox green staining) with diffused FM4-64 staining, while the white arrow indicates a viable cell (negative to Sytox green) with diffused FM4-64 staining.

p-q) ROS accumulation and cell viability for mutants defective in either vacuolar (*vma1, pep3, pep4*) or peroxisomal (*pex6*) function were evaluated in cells grown to late exponential phase in either SCD or SCD_{Cd} liquid media. The fraction of cells exhibiting DHE (h) or PI (i) fluorescence was evaluated by cytofluorimetric analysis. Bars are means ± SDs from two independent experiments.

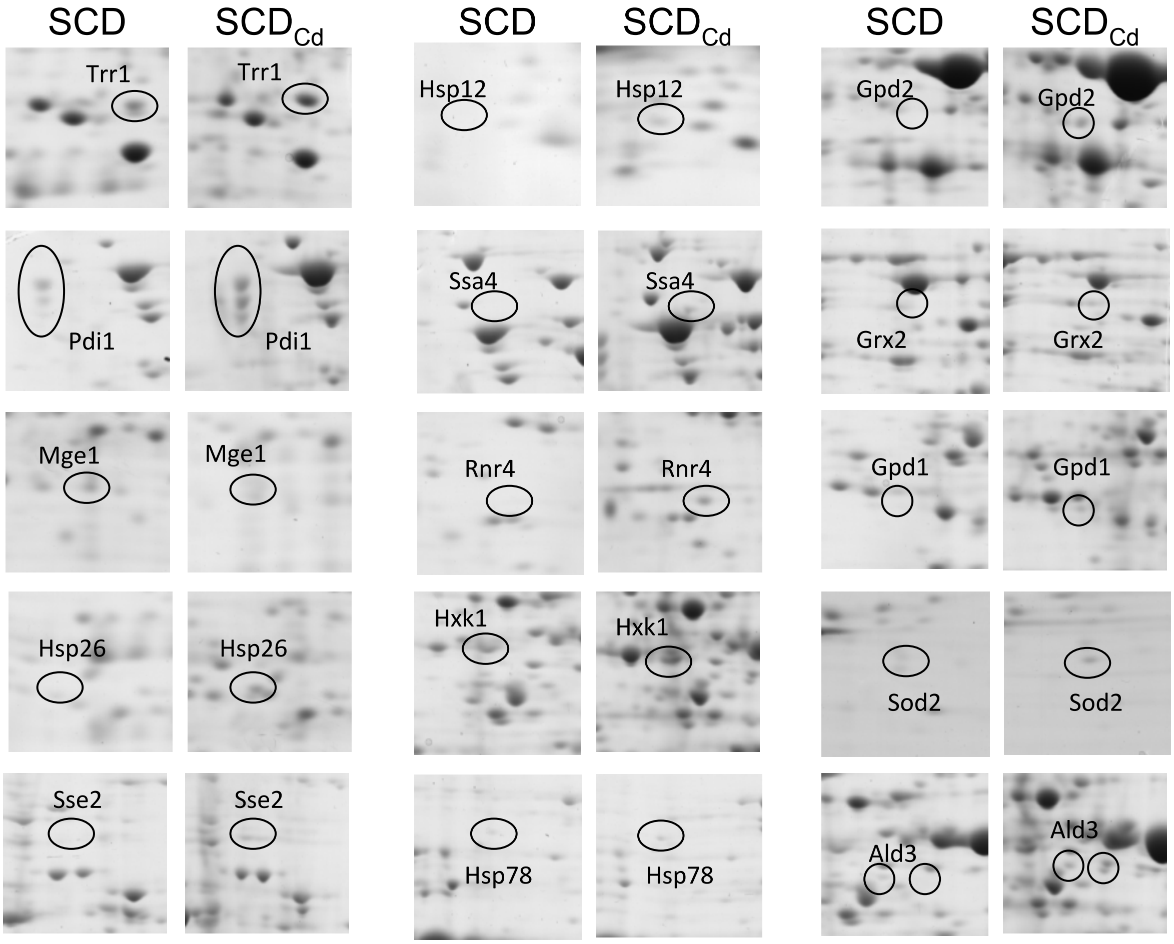

Figure S3 Calcium shortage strongly influences the proteomic profile
Pattern of expression of selected differentially expressed proteins in SCD and SCD_{Cd} media.

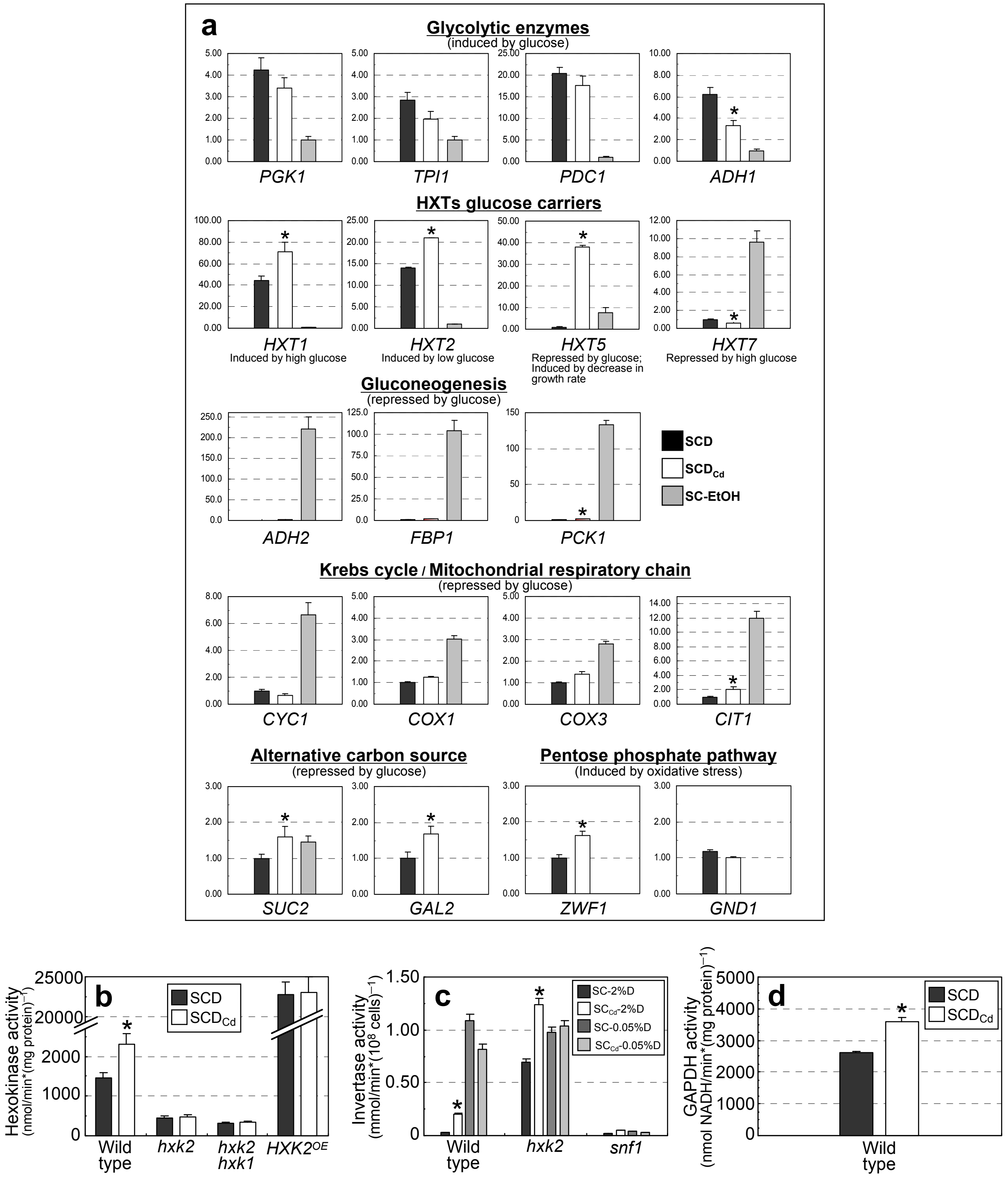

Figure S4. Transcriptional profile of glucose repressed and induced genes during growth under calcium shortage

a) The qRT-PCR analysis was performed on RNA extracted from cells cultivated in SCD, SCD_{Cd} or SC-EtOH media. Values are means ± SDs from at least two independent experiments and were normalized according to the expression level measured under the "basal" growth condition taken as reference (i.e. ethanol for glucose-induced genes, glucose for glucose repressed genes). (*p<0.05, two-tailed t-test).

b-d) Hexokinase, invertase and glyceraldehyde 3-phosphate dehydrogenase (GAPDH) activity in cells cultivated under calcium shortage. W303-1A and isogenic mutant cells were grown in either SCD or SCD_{Cd} media supplemented with 2% glucose. Hexokinase activity (b) of crude extracts was determined as described in Materials and Methods. For Suc2 invertase activity (c), cells growing in SC ethanol medium were harvested and resuspended in either 2% glucose (repressing condition) or 0.05% glucose (inducing conditions). Invertase activity (expressed as µmol of glucose released from saccharose min^{-1})/10^8 cells) was measured in whole cells. *SUC2* transcription is constitutively derepressed in the *hxk2* mutant, whereas is constitutively repressed in the *snf1* null strain. GAPDH activity (d) of crude extracts (expressed as nmol of NADH produced/min*(mg protein)$^{-1}$)) was determined as described in Materials and Methods. The higher overall GAPDH activity measured in cells cultivated in SCD_{Cd} medium is likely a consequence of the increased expression of the Tdh1 isoenzyme (>10-fold, Table SI). The Tdh1 isoform of GAPDH is usually detected in stationary phase cells and its abundance increases in response to stress. Values reported are means ± SDs from at least two independent experiments (*p<0.05, two-tailed t-test).

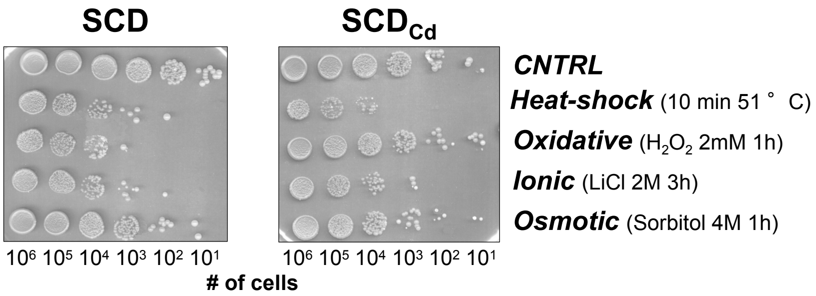

Figure S5 Enhanced resistance to oxidative stress in cells cuktivated under calcium shortage. W303-1A cultivated in either SCD or SCD$_{Cd}$ media were exposed to the indicated stress conditions. Cellular samples were then serially diluted and spotted on YPD plates. Photographs were taken after 48 h incubation at 30 ° C.

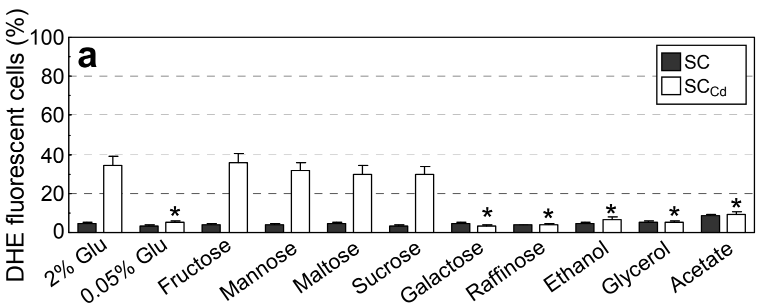

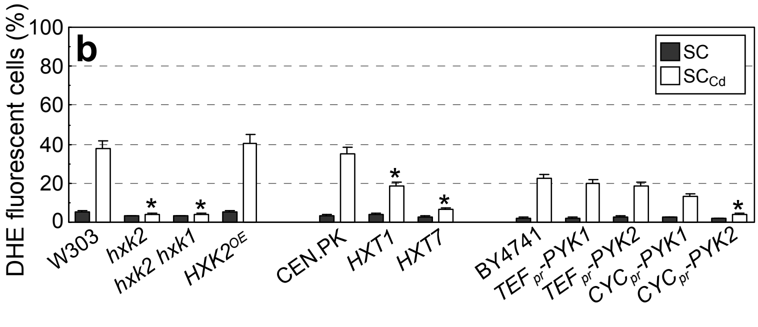

c

Glucose consumption rate in mutants in sugar uptake mechanisms.

Strains	Relative glucose consumption
CEN.PK2-1C	1.000 ± 0.050
hxt(1-17)gal2 TPI$_{pr}$-HXT1::HIS3 [a]	0.470 ± 0.073
hxt(1-17)gal2 TPI$_{pr}$-HXT7::HIS3 [b]	0.144 ± 0.040

The glucose consumption rate was determined in cells grown overnight in either SCD or SCD$_{Cd}$ media and resuspended in medium containing 50 mM glucose at a final density of about 4*10^{6}cells/mL.
Values reported (mean ±SDs from three biological replicates) are relative to glucose consumption rate measured in the wild type strain.
[a] the *hxt(1-17) gal2 HXT1* strain constitutively expresses *HXT1*, a low affinity, high capacity carrier as its only glucose transporter
[b] the *hxt(1-17) gal2 HXT7* strain constitutively expresses *HXT7*, a high affinity carrier, low capacity carrier as its only glucose transporter.

Figure S6 Wild type cells cultivated on non-fermentable carbon sources and strains with constitutive respiratory metabolism do not accumulate ROS under calcium shortage

a) Intracellular ROS accumulation in wild type cells cultivated on various carbon sources under calcium shortage. Values are means ± SDs of two biological replicates (*$p<0.05$, two-tailed *t*-test).

b) Intracellular ROS accumulation in mutants with reduced hexokinase, glucose uptake and pyruvate kinase activity cultivated in either SCD or SCD$_{Cd}$ media. Values are means ± SDs of three biological replicates (*$p<0.05$, two-tailed *t*-test).

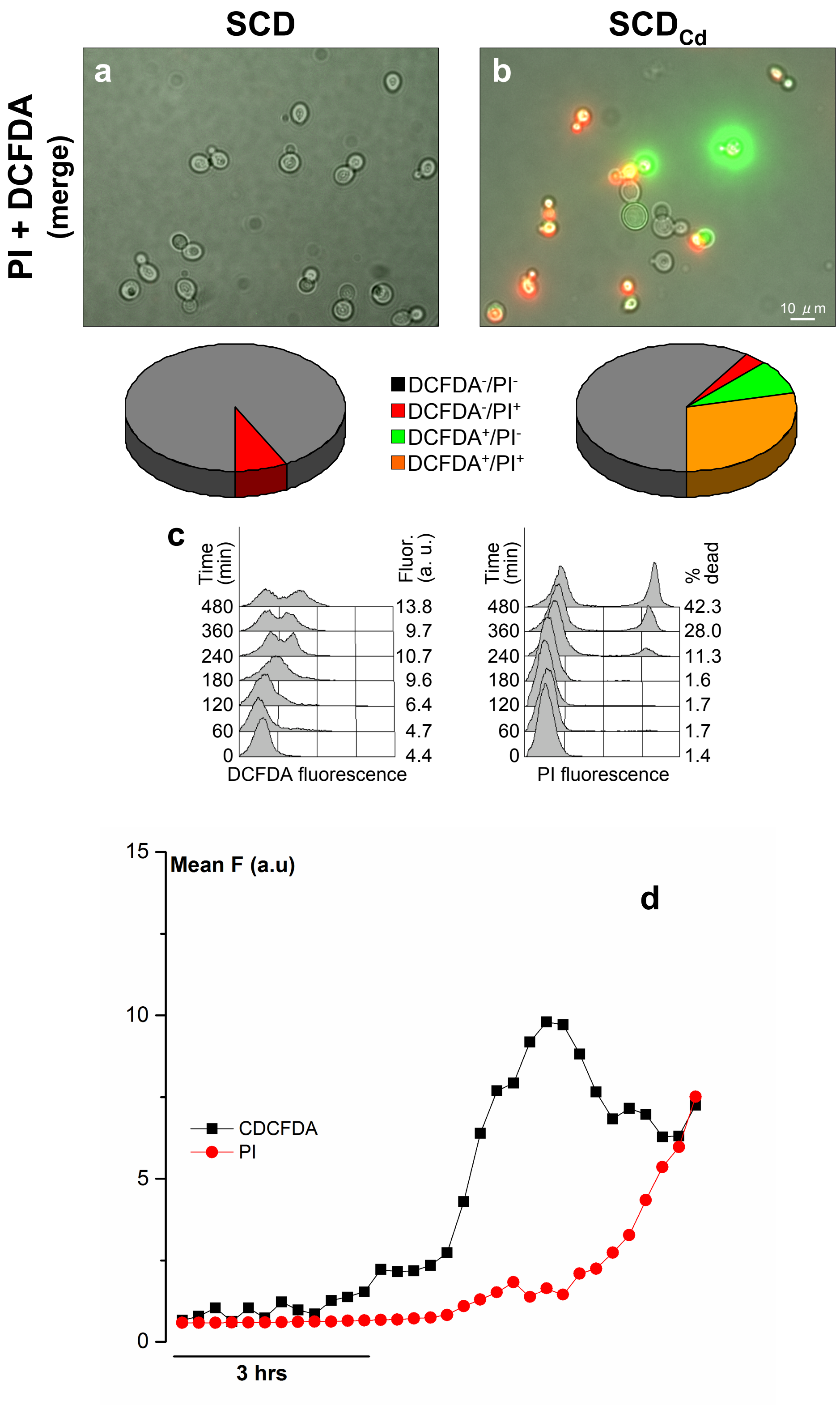

Figure S7 ROS accumulation precedes cell death in calcium-starved yeast cells

a-b) Cells growing in SCD and SCD$_{Cd}$ medium were co-stained with DCFDA and propidium iodide (PI) for the simultaneous detection of ROS accumulation and cell viability. Cells were scored by direct microscopic observation and classified according to their pattern of fluorescence. Representative merged fluorescence microscopic images and the corresponding quantifications are shown. (Means of biological triplicate; n>500 cells).

c) Log-phase cells cultivated in SCD medium were resuspended either in SCD or SCD$_{Cd}$ fresh medium. Intracellular ROS accumulation (left panel) and cell viability (right panel) were evaluated by cytofluorimetric analysis. Data representative from two biological replicate are shown.

d) A global fluorescence analysis in a population of calcium-starved cells stained with DCFD/PI was performed by time-lapse microscopy. Cells were shifted in SCD$_{Cd}$ medium at time 0 and DCFDA and PI fluorescence was monitored every 15 min.

(to be continued)

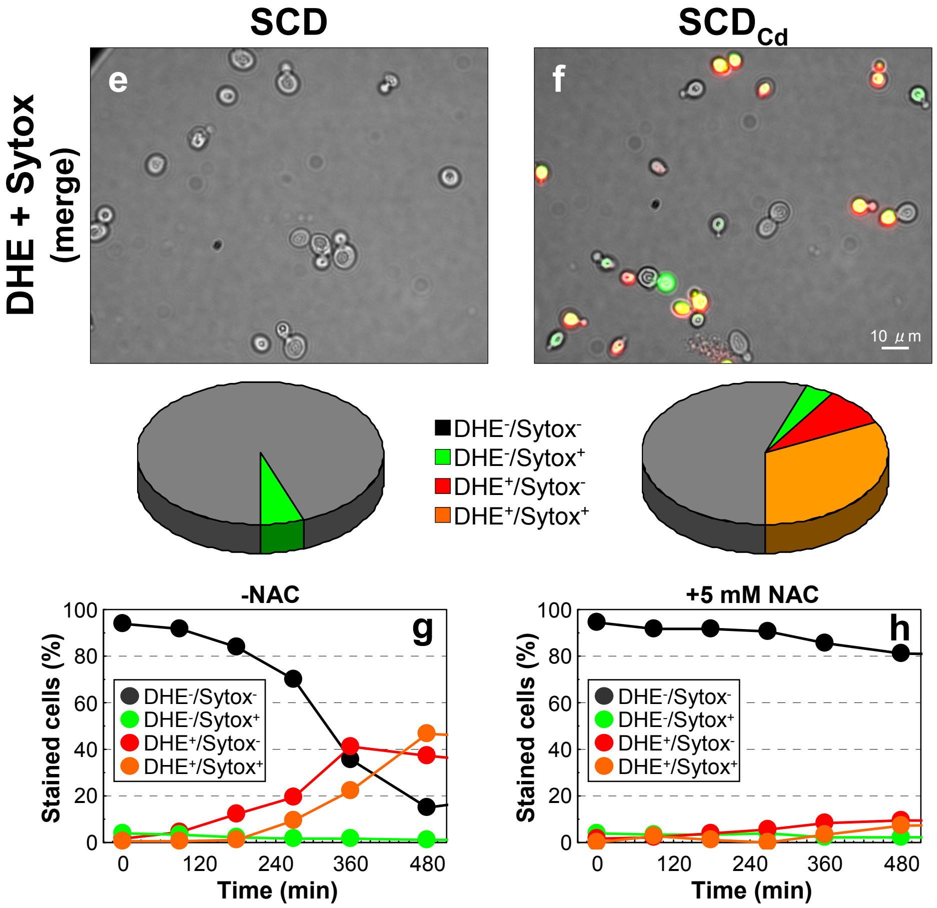

Figure S7 ROS accumulation precedes cell death in calcium-starved yeast cells
(continues)

e-f) Cells growing in SCD and SCD$_{Cd}$ medium were co-stained with DHE and Sytox Green for the simultaneous detection of ROS accumulation and cell viability. Cells were scored by direct microscopic observation and classified according to their pattern of fluorescence. Representative merged fluorescence microscopic images and the corresponding quantifications are shown. (Means of biological duplicate; n>500 cells).

g-h) SCD-cultivated cells were transferred to SCD$_{Cd}$ medium (in either the presence (right) or absence (left) of 5 mM NAC). At the indicated time points, intracellular ROS accumulation and cell viability were evaluated by direct observation of cells co-stained with DHE and Sytox Green. Data representative from biological duplicates are shown.

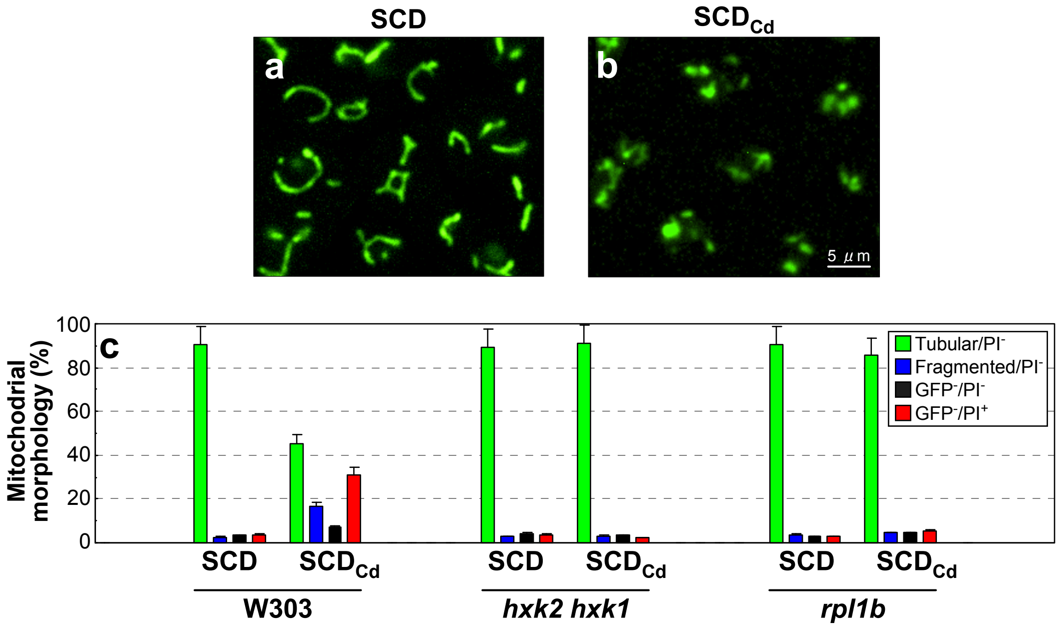

Fig. S8. No alterations in mitochondrial morphology under calcium shortage are detected in strains with constitutive respiratory metabolism (hxk2 hxk1 cells) or in slow-growing mutants with reduced protein synthesys rate (rpl1b cells).

a-b) Wild type mtGFP-expressing cells were cultivated to log-phase in SCD medium, harvested and transferred in either SCD or SCD_{Cd} fresh media. Selected fluorescence microscopy images (acquired 6 hours after the shift) showing alterations in mitochondrial morphology are reported.

c) Classification of wild type and mutant cells exponentially growing in SCD_{Cd} media according to the morphology of their mitochondria and their viability. The analysis was performed by direct microscopic observation of propidium iodide-stained cells. Values are means ± SDs of two biological replicates. GFP-negative (GFP-) cells lacked any detectable mtGFP signal.

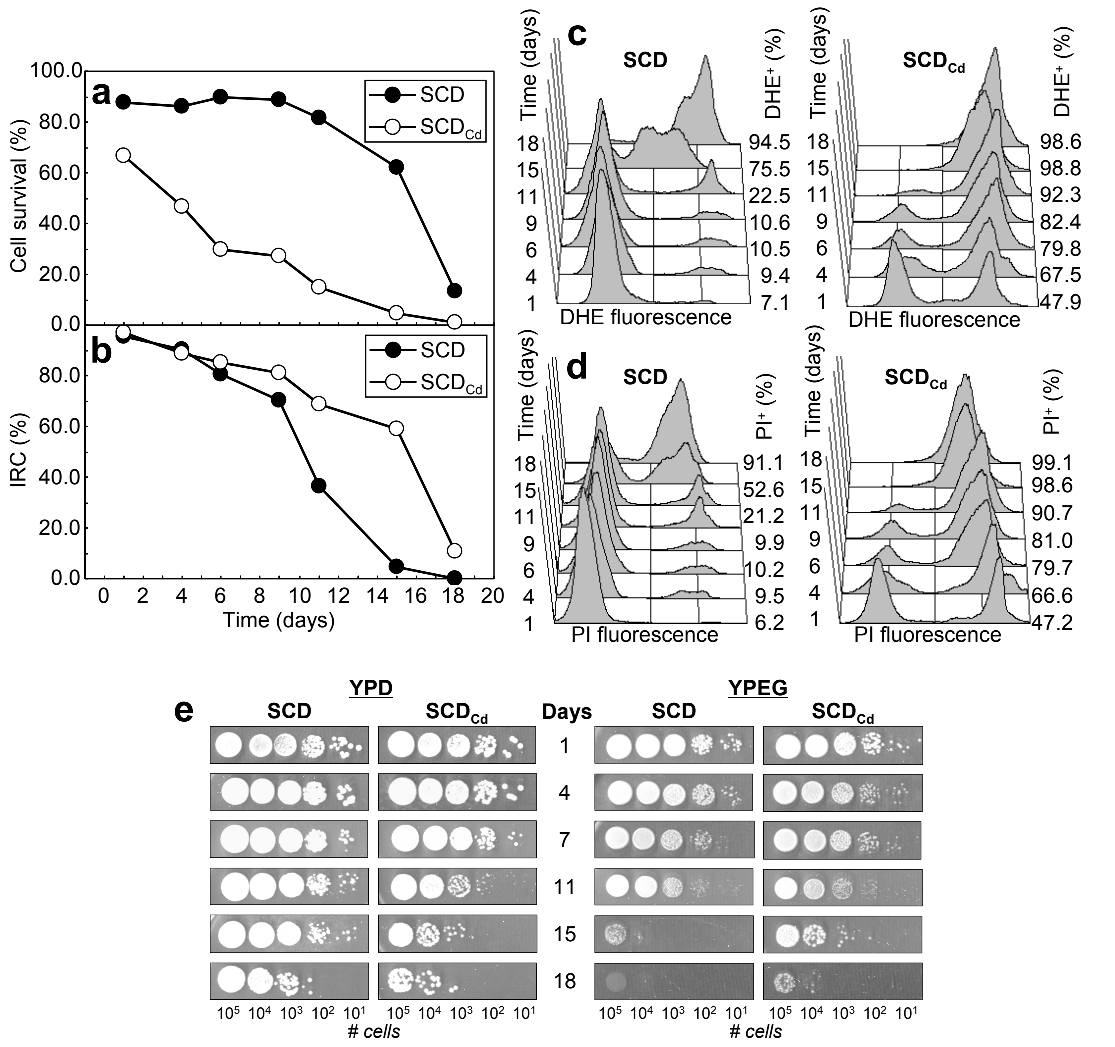

Figure S9 Calcium shortage shortens yeast chronological life span

a) Loss of cell viability in aging yeast cultures. At each time-point, viability was determined by direct count of at least 500 cells stained with methylene blue. Clonogenic assays (i.e. counting CFUs on YPD plates) gave similar results (not shown).

b) Index of Respiratory competence (IRC (here defined as the percentage of viable cells within a population which are competent to respire). Identical amounts of diluted cell coltures were plated on glucose (YPD) and ethanol/glycerol (YPEG) plates. IRC was calculated as the ratio between the colonies grown on YPEG and YPD.

c) Intracellular ROS accumulation. The fraction of cells positive to dihydroethidium (DHE) staining was scored by cytofluorimetric analysis.

d) Loss of membrane integrity. The fraction of cells positive to propidium iodide staining (PI) was evaluated by cytofluorimetric analysis or by direct observation on at least 1000 cells (with consistent result).

e) Loss of mitocondrial functionality in aging yeast cultures under calcium shortage. Cellular suspensions of stationary phase cultures were serially diluted and spotted on YP plates supplemented with either glucose (YPD) or ethanol/glycerol (YPEG). Plates were photographed after 48h at 30° C.

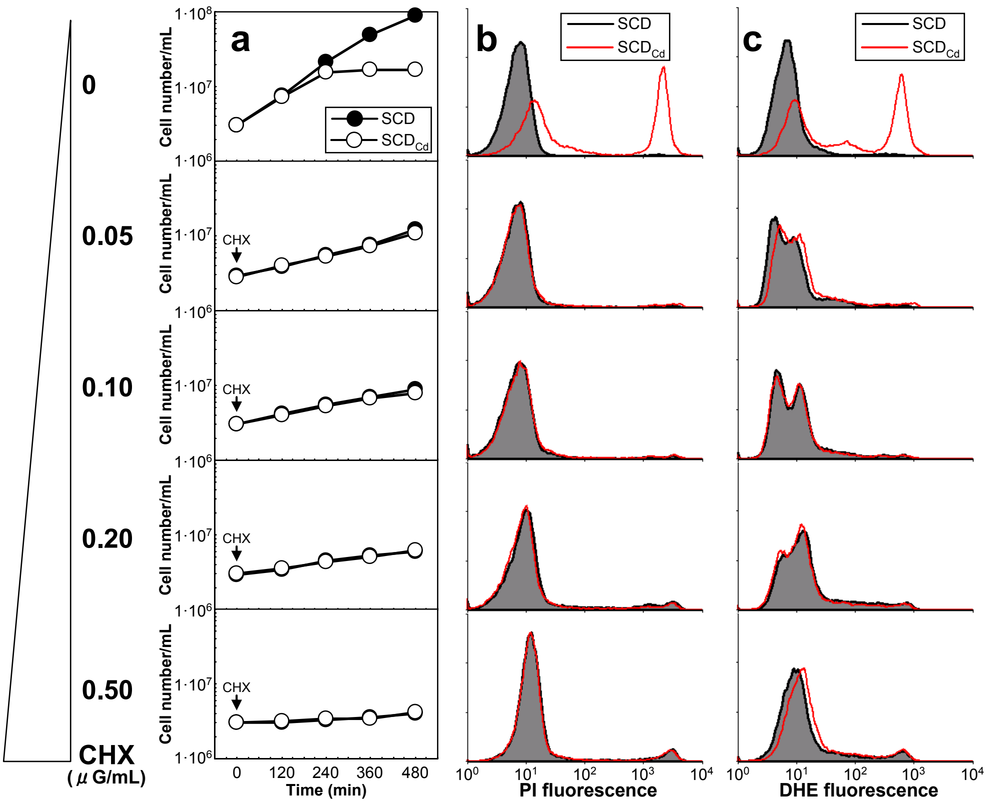

Fig. S10 Inhibition of protein synthesis by cycloheximide prevents the insurgence of calcium shortage related effects.
W303-1A cells were cultivated in SCD media (~5*10^6 cells/mL). At time point 0, the culture was splitted and cells were resuspended either in SCD or SCD$_{Cd}$ media containing cycloheximide at the indicated concentrations.

a) Growth kinetic after the shift in the presence of cycloheximide. **b, c)** Loss of membrane integrity (PI staining) and ROS accumulation (DHE staining) as evaluated by cytofluorimetric analysis on cellular samples collected 480min after the shift.

Note 1: calcium shortage-related phenotypes do not appear even 16-24h after the shift in the presence of cycloheximide.

Note 2: the profiles obtained by cells treated with cycloheximide (0.05-0.20 uG/mL) after DHE staining apparently suggest the existence of a subcellular population with increased ROS content, both in SCD or SCD$_{Cd}$ media (panel C). Nonetheless, cells maintain full viability.

Note 3: cycloheximide concentration higher than 1uG/mL completely block the progression of the cell division cycle

Table SI. Growth parameters in SC and SC_{Cd} media supplemented with different carbon sources,

Strain	***Medium***	***MDT*** ***(min)***	***Average cell Volume*** ***(fL)***	***Unbudded cells*** ***(%)***	***G1 phase cells*** ***(%)***
**W303-1A**	***SC-Glucose***	96 ± 3	54.9 ± 3	31 ± 3	26 ± 3
	SC_{Cd}-Glucose	257 ± 26	45.3 ± 3	30 ± 2	15 ± 3
	SC-Fructose	111 ± 6	51.7± 2	28 ± 2	23 ± 3
	SC_{Cd}-Fructose	267 ± 17	47.2 ± 3	27 ± 3	14 ± 4
	SC-Mannose	137 ± 8	49.5 ± 1	29 ± 4	25 ± 4
	SC_{Cd}-Mannose	198 ± 24	47.6 ± 3	28 ± 5	15 ± 3
	SC-Sucrose	135 ± 10	49.5 ± 2	27 ± 1	23 ± 3
	SC_{Cd}-Sucrose	315 ± 23	44.6 ± 3	29 ± 2	16 ± 2
	SC-Galactose	108 ± 3	48.5 ± 4	38 ± 2	33 ± 1
	SC_{Cd}-Galactose	111 ± 10	47.7 ± 3	40 ± 3	34 ± 3
	SC-Ethanol	213 ± 15	37.5 ± 1	48 ± 2	42 ± 3
	SC_{Cd}-Ethanol	212 ± 17	37.7 ± 1	48 ± 2	41 ± 2
	SC-0.5% Glucose	101 ± 4	52.8 ± 4	29 ± 2	25 ± 4
	SC_{Cd}-0.5%Glucose	228 ± 14	43.3 ± 3	32 ± 3	14 ± 3
	SC-0.05% Glucose	145 ± 8	44.2 ± 2	35 ± 4	30 ± 5
	SC_{Cd}-0.05% Glucose	152 ± 10	45.3 ± 1	36 ± 3	28 ± 6
hxk2	***SC-Glucose***	139 ± 12	34 ± 2	43 ± 3	
	SC_{Cd}-Glucose	156 ± 8	36 ± 2	38 ± 4	
hxk2 hxk1	***SC-Glucose***	156 ± 5	25 ± 1	41 ± 2	
	SC_{Cd}-Glucose	148 ± 12	26 ± 1	44 ± 2	

MDT = mass doubling time (min).

Growth parameters were monitored during growth at 30°C in SC medium supplemented with the indicated carbon sources (all at 2% w/v except where otherwise stated). The percentage of unbudded cells was scored by direct microscopic observation. The fraction of cells in G1 phase (i. e. with 1c DNA content) was determined by cytofluorimetric analysis. Cell volumes were determined with a Coulter particle analyzer. Values reported are means ± standard deviations of at least 3 independent experiments.

Table SIIa - Proteins differentially expressed during growth under calcium shortage

Systematic name	NAME	FOLD CHANGE (SCD_{Cd}/SCD)	Description
YCR088W	**ABP1**	6.37	Actin-binding protein of the cortical actin cytoskeleton; important for activation of the Arp2/3 complex that plays a key role actin in cytoskeleton organization
YLR153C	**ACS2**	2.90	Acetyl-coA synthetase isoform which, along with Acs1p, is the nuclear source of acetyl-coA for histone acetylation; required for growth on glucose; expressed under anaerobic conditions
YOL086C	**ADH1**	-1.78	Alcohol dehydrogenase, the major enzyme responsible for converting acetaldehyde to ethanol, the last step in the glycolytic pathway; gene transcription is repressed when cells are grown on non-fermentable carbon sources
YJR105W	**ADO1**	404.56	Adenosine kinase, required for the utilization of S-adenosylmethionine (AdoMet)
YMR169C	**ALD3**	2.86	Cytoplasmic aldehyde dehydrogenase, involved in ethanol oxidation and beta-alanine biosynthesis: expression is induced by stress and glucose repressed
YPL061W	**ALD6**	1.93	Cytosolic aldehyde dehydrogenase, required for conversion of acetaldehyde to acetate; constitutively expressed; locates to the mitochondrial outer surface upon oxidative stress
YBR149W	**ARA1**	466.66	NADP+ dependent arabinose dehydrogenase, involved in carbohydrate metabolism
YOL058W	**ARG1**	2.20	Arginosuccinate synthetase
YKL007W	**CAP1**	7.20	Alpha subunit of the capping protein (CP) heterodimer (Cap1p and Cap2p) which binds to the barbed ends of actin filaments preventing further polymerization; localized predominantly to cortical actin patches
YOR133W	**EFT1**	-3.93	Elongation factor 2 (EF-2), catalyzes ribosomal translocation during protein synthesis;
YGR254W	**ENO1**	5.01	Enolase I, a phosphopyruvate hydratase that catalyzes the conversion of 2-phosphoglycerate to phosphoenolpyruvate during glycolysis and the reverse reaction during gluconeogenesis; expression repressed in response to glucose; protein abundance increases i
YHR174W	**ENO2**	3.03	Enolase II, a phosphopyruvate hydratase that catalyzes the conversion of 2-phosphoglycerate to phosphoenolpyruvate during glycolysis and the reverse reaction during gluconeogenesis; expression induced in response to glucose
YPL028W	**ERG10**	661.51	Acetyl-CoA C-acetyltransferase (acetoacetyl-CoA thiolase), cytosolic enzyme that transfers an acetyl group from one acetyl-CoA molecule to another, forming acetoacetyl-CoA, the first step in the biosynthesis of mevalonate, which is required for the biosyn
YML126C	**ERG13**	2.17	3-hydroxy-3-methylglutaryl-CoA (HMG-CoA) synthase, catalyzes the formation of HMG-CoA from acetyl-CoA and acetoacetyl-CoA, the second step in mevalonate biosynthesis
YML008C	**ERG6**	2.29	Delta(24)-sterol C-methyltransferase, converts zymosterol to fecosterol in the ergosterol biosynthetic pathway; localized to lipid particles, the plasma membrane-associated endoplasmic reticulum, and the mitochondrial outer membrane
YEL047C	**FRD1**	1.84	Soluble fumarate reductase
YOR375C	**GDH1**	-1.62	NADP(+)-dependent glutamate dehydrogenase, synthesizes glutamate from ammonia and alpha-ketoglutarate; expression regulated by nitrogen and carbon sources
YCL040W	**GLK1**	3.78	Glucokinase, catalyzes the phosphorylation of glucose at C6 in the first irreversible step of glucose metabolism; expression is glucose repressed
YDL022W	**GPD1**	87.15	NAD-dependent glycerol-3-phosphate dehydrogenase; key enzyme of glycerol synthesis, essential for growth under osmotic stress; expression regulated by HOG pathway
YOL059W	**GPD2**	315.14	NAD-dependent glycerol 3-phosphate dehydrogenase; homolog of Gpd1p, expression is controlled by an oxygen-independent signaling pathway required to regulate metabolism under anoxic conditions; located in cytosol and mitochondria; constitutively active but
YCL035C	**GRX1**	130.38	Hydroperoxide and superoxide-radical responsive heat-stable glutathione-dependent disulfide oxidoreductase, protects cells from oxidative damage
YDR454C	**GUK1**	-3.72	Guanylate kinase, converts GMP to GDP; required for growth and mannose outer chain elongation of cell wall N-linked glycoproteins
YLR192C	**HCR1**	1.80	Dual function protein involved in translation initiation as a substoichiometric component (eIF3j) of translation initiation factor 3 (eIF3) and required for processing of 20S pre-rRNA; binds to eIF3 subunits Rpg1p and Prt1p and 18S rRNA
YER062C	**HOR2**	240.87	One of two redundant DL-glycerol-3-phosphatases involved in glycerol biosynthesis; induced in response to hyperosmotic stress and oxidative stress, and during the diauxic transition
YFL014W	**HSP12**	197.66	Plasma membrane localized protein that protects membranes from desiccation; induced by heat shock, oxidative stress, osmostress, stationary phase entry, glucose depletion, oleate and alcohol;
YBR072W	**HSP26**	2.81	Small heat shock protein (sHSP) with chaperone activity; forms hollow, sphere-shaped oligomers that suppress unfolded proteins aggregation; not expressed in unstressed cells
YDR258C	**HSP78**	2.15	Oligomeric mitochondrial matrix chaperone that cooperates with Ssc1p in mitochondrial thermotolerance after heat shock; able to prevent the aggregation of misfolded proteins as well as resolubilize protein aggregates

Systematic name	NAME	FOLD CHANGE (SCD_{Cd}/SCD)	Description
YFR053C	**HXK1**	2.57	Hexokinase isoenzyme 1, a cytosolic protein that catalyzes phosphorylation of glucose during glucose metabolism; expression is glucose repressed and is highest during growth on non-glucose carbon sources
YJL153C	**INO1**	173.12	Inositol 1-phosphate synthase, involved in synthesis of inositol phosphates and inositol-containing phospholipids;
YJL034W	**KAR2**	3.39	ATPase involved in protein import into the ER, also acts as a chaperone to mediate protein folding in the ER and may play a role in ER export of soluble proteins; regulates the unfolded protein response via interaction with Ire1p
YOR232W	**MGE1**	163.59	Mitochondrial matrix cochaperone; acts as a nucleotide release factor for Ssc1p in protein translocation and folding
YKL142W	**MRP8**	4.55	Protein of unknown function
YHR179W	**OYE2**	71.18	Conserved NADPH oxidoreductase; may be involved in sterol metabolism, oxidative stress response and programmed cell death; protein abundance increases in response to DNA replication stress
YCL043C	**PDI1**	2.76	Protein disulfide isomerase, resident in the ER lumen, essential for the formation of disulfide bonds in secretory and cell-surface proteins
YOR361C	**PRT1**	-3.77	eIF3b subunit of the core complex of translation initiation factor 3 (eIF3); part of a subcomplex (Prt1p-Rpg1p-Nip1p) that stimulates binding of mRNA and tRNA(i)Met to ribosomes
YGR253C	**PUP2**	6.84	Alpha 5 subunit of the 20S proteasome involved in ubiquitin-dependent catabolism
YPR191W	**QCR2**	100.00	Subunit 2 of the ubiquinol cytochrome-c reductase complex (Commplex III), which is a component of the mitochondrial inner membrane electron transport chain; gene transcription is regulated by Hap1p, Hap2p/Hap3p, and heme
YCR028C-A	**RIM1**	102.51	Single-stranded DNA-binding protein essential for mitochondrial genome maintenance; involved in mitochondrial DNA replication
YGR180C	**RNR4**	310.00	Ribonucleotide-diphosphate reductase (RNR), small subunit; the RNR complex catalyzes the rate-limiting step in dNTP synthesis and is regulated by DNA replication and DNA damage checkpoint pathways via localization of the small subunits
YKR057W	**RPS21A**	2.81	Protein component of the small (40S) ribosomal subunit
YDR502C	**SAM2**	2.96	S-adenosylmethionine synthetase
YFL045C	**SEC53**	2.56	Phosphomannomutase, involved in synthesis of GDP-mannose and dolichol-phosphate-mannose; required for folding and glycosylation of secretory proteins in the ER lumen
YHR008C	**SOD2**	1.96	Mitochondrial superoxide dismutase, protects cells against oxygen toxicity
YPR069C	**SPE3**	-2.38	Spermidine synthase, involved in biosynthesis of spermidine and also in biosynthesis of pantothenic acid
YER103W	**SSA4**	275.42	Heat shock protein that is highly induced upon stress; plays a role in SRP-dependent cotranslational protein-membrane targeting and translocation; member of the HSP70 family; cytoplasmic protein that concentrates in nuclei upon starvation
YBR169C	**SSE2**	60.48	Member of the heat shock protein 70 (HSP70) family; involved in protein folfing
YJL052W	**TDH1**	941.87	Glyceraldehyde-3-phosphate dehydrogenase, isozyme 1, involved in glycolysis and gluconeogenesis; Tdh1p is primarily detected during stationary phase; protein abundance increases in response to several stress conditions (DNA replication stress, reductive
YMR146C	**TIF34**	-1.65	eIF3i subunit of the core complex of translation initiation factor 3 (eIF3)
YPR041W	**TIF5**	59.72	Translation initiation factor eIF5
YDR353W	**TRR1**	2.03	Cytoplasmic thioredoxin reductase; key regulatory enzyme that determines the redox state of the thioredoxin system, which protects cells against both oxidative and reductive stress
YML028W	**TSA1**	4.96	Thioredoxin peroxidase; acts as both a ribosome-associated and free cytoplasmic antioxidant
YEL021W	**URA3**	-4.09	Orotidine-5'-phosphate (OMP) decarboxylase, catalyzes the sixth enzymatic step in the de novo biosynthesis of pyrimidines
YOR230W	**WTM1**	6.03	Transcriptional modulator involved in regulation of meiosis, silencing, and expression of RNR genes; required for nuclear localization of the ribonucleotide reductase small subunit Rnr2p and Rnr4p;

Table SIIb - GeneCodis Functional class analysis of proteins showing a differential expression under calcium shortage

Id	GO Biological Process ID	Description	Hyp	Hyp_corrected	Genes	Support	Reference Support	Reference size
3	GO:0006006	glucose metabolic process (BP)	5.45E-04	5.02E-03	*HXK1,GLK1,TDH1*	3	22	7109
4	GO:0006950	response to stress (BP)	1.29E-05	5.95E-04	*HSP26,HSP12,HOR2,GPD1,KAR2,SSA4,SSE2,HSP78*	8	150	7109
11	GO:0008152	metabolic process (BP)	1.22E-04	2.10E-03	*ALD3,HXK1,ERG6,HOR2,ERG10,OYE2,URA3,SEC53,ALD6,ERG13,ACS2*	11	394	7109
12	GO:0006096	glycolysis (BP)	9.01E-07	6.22E-05	*HXK1,ENO1,GLK1,ENO2,TDH1*	5	25	7109
16	GO:0055114	oxidation-reduction process (BP)	1.13E-08	1.56E-06	*ADH1,ALD3,ARA1,RNR4,YEL047C,SOD2,GPD1,TRR1,OYE2,GDH1,TSA1,ALD6,GPD2,TDH1*	14	284	7109
17	GO:0006094	gluconeogenesis (BP)	2.95E-04	3.70E-03	*ENO1,ENO2,TDH1*	3	18	7109
18	GO:0030150	protein import into mitochondrial matrix (BP)	9.51E-03	3.65E-02	*MGE1,HSP78*	2	20	7109
22	GO:0042026	protein refolding (BP)	2.05E-04	2.82E-03	*MGE1,SSE2,HSP78*	3	16	7109
23	GO:0034605	cellular response to heat (BP)	8.60E-03	3.49E-02	*HSP12,HSP78*	2	19	7109
28	GO:0034599	cellular response to oxidative stress (BP)	1.41E-03	1.14E-02	*HSP12,TRR1,GRX1,TSA1*	4	66	7109
30	GO:0006412	translation (BP)	8.80E-03	3.47E-02	*EFT1,TIF5,HCR1,RPS21A,TIF34,PRT1*	6	242	7109
33	GO:0006457	protein folding (BP)	7.13E-05	1.41E-03	*HSP26,MGE1,SSA4,SSE2,PDI1,TSA1*	6	96	7109
38	GO:0005975	carbohydrate metabolic process (BP)	5.31E-03	3.49E-02	*HXK1,GLK1,GPD1,GPD2*	4	95	7109
41	GO:0006021	inositol biosynthetic process (BP)	1.49E-02	4.02E-02	*INO1*	1	2	7109
50	GO:0046168	glycerol-3-phosphate catabolic process (BP)	5.45E-05	1.51E-03	*GPD1,GPD2*	2	2	7109
52	GO:0006116	NADH oxidation (BP)	4.53E-05	1.56E-03	*ADH1,GPD1,GPD2*	3	10	7109
53	GO:0006072	glycerol-3-phosphate metabolic process (BP)	3.24E-04	3.44E-03	*GPD1,GPD2*	2	4	7109
55	GO:0044262	cellular carbohydrate metabolic process (BP)	6.90E-03	4.33E-02	*ARA1,HOR2*	2	17	7109
58	GO:0006696	ergosterol biosynthetic process (BP)	6.24E-04	5.38E-03	*ERG6,ERG10,ERG13*	3	23	7109
59	GO:0016126	sterol biosynthetic process (BP)	1.70E-02	4.51E-02	*ERG6,ERG13*	2	27	7109
65	GO:0001302	replicative cell aging (BP)	2.96E-03	2.15E-02	*SOD2,TSA1,ACS2*	3	39	7109
66	GO:0006413	translational initiation (BP)	4.55E-04	4.49E-03	*TIF5,HCR1,TIF34,PRT1*	4	49	7109
72	GO:0006616	SRP-dependent cotranslational protein targeting to membrane, translocation (BP)	2.36E-03	1.81E-02	*KAR2,SSA4*	2	10	7109
77	GO:0045454	cell redox homeostasis (BP)	6.54E-05	1.50E-03	*TRR1,PDI1,GRX1,TSA1*	4	30	7109
80	GO:0006085	acetyl-CoA biosynthetic process (BP)	1.49E-02	4.02E-02	*ACS2*	1	2	7109
82	GO:0032889	regulation of vacuole fusion, non-autophagic (BP)	4.04E-03	2.78E-02	*ENO1,ENO2*	2	13	7109
87	GO:0043458	ethanol biosynthetic process involved in glucose fermentation to ethanol (BP)	1.49E-02	4.02E-02	*ADH1*	1	2	7109
94	GO:0033194	response to hydroperoxide (BP)	7.46E-03	3.12E-02	*TSA1*	1	1	7109
95	GO:0042262	DNA protection (BP)	7.46E-03	3.12E-02	*TSA1*	1	1	7109
102	GO:0001320	age-dependent response to reactive oxygen species involved in chronological cell aging (BP)	1.49E-02	4.02E-02	*SOD2*	1	2	7109
108	GO:0044205	'de novo' UMP biosynthetic process (BP)	7.46E-03	3.12E-02	*URA3*	1	1	7109
112	GO:0008295	spermidine biosynthetic process (BP)	1.49E-02	4.02E-02	*SPE3*	1	2	7109
113	GO:0009186	deoxyribonucleoside diphosphate metabolic process (BP)	1.49E-02	4.02E-02	*RNR4*	1	2	7109
119	GO:0051693	actin filament capping (BP)	1.49E-02	4.02E-02	*CAP1*	1	2	7109
121	GO:0006973	intracellular accumulation of glycerol (BP)	7.46E-03	3.12E-02	*GPD1*	1	1	7109
122	GO:0000052	citrulline metabolic process (BP)	7.46E-03	3.12E-02	*ARG1*	1	1	7109
123	GO:0000053	argininosuccinate metabolic process (BP)	7.46E-03	3.12E-02	*ARG1*	1	1	7109
124	GO:0071470	cellular response to osmotic stress (BP)	7.46E-03	3.12E-02	*HSP12*	1	1	7109
125	GO:0007009	plasma membrane organization (BP)	1.49E-02	4.02E-02	*HSP12*	1	2	7109
127	GO:0006163	purine nucleotide metabolic process (BP)	1.49E-02	4.02E-02	*GUK1*	1	2	7109
128	GO:0006167	AMP biosynthetic process (BP)	1.49E-02	4.02E-02	*ADO1*	1	2	7109
129	GO:0046037	GMP metabolic process (BP)	7.46E-03	3.12E-02	*GUK1*	1	1	7109
134	GO:0051188	cofactor biosynthetic process (BP)	7.46E-03	3.12E-02	*RNR4*	1	1	7109
137	GO:0019483	beta-alanine biosynthetic process (BP)	1.49E-02	4.02E-02	*ALD3*	1	2	7109

Table SIIc - REVIGO-filtered non-redundant functional categories for proteins showing a differential expression under calcium shortage

term_ID	description	frequencyInDb	log10pvalue	uniqueness	dispensability	representative
GO:0006096	glycolytic process	1.88%	-6.0453	0.664	0	glycolytic process
GO:0006116	NADH oxidation	1.51%	-4.3439	0.614	0.193	glycolytic process
GO:0046168	glycerol-3-phosphate catabolic process	0.27%	-4.2636	0.773	0.29	glycolytic process
GO:0008295	spermidine biosynthetic process	1.75%	-1.8268	0.726	0.481	metabolism
GO:0009186	deoxyribonucleoside diphosphate metabolic process	0.13%	-1.8268	0.764	0.276	metabolism
GO:0055114	oxidation-reduction process	12.63%	-7.9469	0.827	0.149	glycolytic process
GO:0000052	citrulline metabolic process	0.61%	-2.1273	0.748	0.406	metabolism
GO:0044205	'de novo' UMP biosynthetic process	0.13%	-2.1273	0.651	0.337	metabolism
GO:0006696	ergosterol biosynthetic process	0.27%	-3.2048	0.656	0.159	metabolism
GO:0006006	glucose metabolic process	0.27%	-3.2636	0.745	0.446	glycolytic process
GO:0044262	cellular carbohydrate metabolic process	8.33%	-2.1612	0.805	0.489	glycolytic process
GO:0006950	response to stress	3.36%	-4.8894	0.782	0	response to stress
GO:0034599	cellular response to oxidative stress	0.13%	-2.8508	0.694	0.466	response to stress
GO:0008152	metabolic process	80.78%	-3.9136	0.991	0	metabolism
GO:0006457	protein folding	0.27%	-4.1469	0.852	0.041	protein folding
GO:0006412	translation	9.95%	-2.0555	0.746	0.487	protein folding
GO:0006413	translational initiation	0.13%	-3.342	0.811	0.32	protein folding
GO:0042026	protein refolding	0.13%	-3.6882	0.858	0.32	protein folding
GO:0005975	carbohydrate metabolic process	15.46%	-2.2749	0.906	0.053	glycolytic process
GO:0006616	SRP-dependent cotranslational protein targeting to membrane, translocation	0.13%	-2.6271	0.754	0.061	protein folding
GO:0045454	cell redox homeostasis	4.12%	-4.1844	0.666	0.09	cell redox homeostasis
GO:0001302	replicative cell aging	51.48%	-2.5287	0.616	0.167	cell redox homeostasis

Table SIIIA. Variation of intracellular metabolites between growth in SC and SC $_{Cd}$ media.

Metabolite	***fold change SC $_{Cd}$/SC***			
	2% glucose	***0.05% glucose***	***2% galactose***	***2% EtOH***
Glucose-6P	green	grey		grey
Fructose-6P	green	grey		grey
Fructose-1,6BP	green	grey		grey
Pyruvate	green	grey		grey
ATP	green	grey		grey
ADP	green	grey		grey
AMP	green	grey		grey
GSH $_{Tot}$	red	red		grey
Valine	red	grey	grey	
Oleic acid	red	grey	grey	
Citric acid	red	grey	grey	
Stearic acid	red	grey	grey	
Palmitic acid	red	grey	grey	
Alanine	grey	red	red	
Leucine	green	grey	grey	
Norleucine	green	grey	grey	
Methionine	green	grey	grey	
Glycine	green	grey	red	
Glutamic acid	green	grey	green	
Serine	green	grey	grey	
Lysine	green	grey	grey	
Proline	green	grey	green	
Aspartic acid	green	grey	green	
Glycine	grey	grey	red	
Threonine	grey	grey	grey	
Ornithine	grey	grey	grey	
Succinate	grey	grey	green	
Lactic acid	grey	grey	grey	
Palmitoleic acid	grey	grey	grey	
Lauric acid	grey	grey	green	
Myristic acid	grey	grey	green	

(red) = increased level in SC medium

(green) = decreased level in SC $_{Cd}$ medium

(grey) = no change

(white) = Not Assayed

Table SIIIB-List of identified metabolites

Abbreviation	Name	Abbreviation	Name
2-AB	2-aminobutyrate	LAC	Lactate
2HIB	2-hydroxyisobutyrate	LAU	Lauric acid
2PG	2-phosphoglycerate	LEU	Leucine
ADI	Adipate	LLCT	Cystathionine
AKB	2-oxobutyrate	LYS	Lysine
AKG	α-ketoglutarate	MAL	Malate
ALA	Alanine	MET	Methionine
AMA	L-2-aminoadipate	MYR	Myristate
ASER	O-actyl-L-serine	NADP	NADP
ASN	Asparagine	NAGLU	N-acetylglutamate
ASP	Aspartate	OAA	Oxalacetate
CAC	Cys-aconitate	OLC	Oleic acid
CIT	Citrate	ORN	Ornithine
CITC	Citraconate	PAL	Palmitate
CITM	Citramalate	PALO	Palmitoleic acid
CUM	Cumarate	PGLU	Pyroglutamate
CYS	Cysteine	PHE	Phenylalanine
DAMA	D-2-aminoadipate	PHT	Phtalate
FUM	Fumavate	PIME	Pimelate
GLN	Glutamine	PRO	Proline
GLU	Glutamate	PYR	Pyruvate
GLUT	Glutarate	SER	Serine
GLY	Glycine	SUC	Succinate
HIS	Histidine	THR	Threonine
HPRO	Trans-4-hydroxyproline	TRP	Triptophane
ICI	Isocitrate	TYR	Tyrosine
ILE	Isoleucine	VAL	Valine
IPPMAL	Isopropylmalate		

Supplementary Movie 1. ROS accumulation precedes cell death in calcium-starved yeast cells

A global fluorescence analysis in a population of calcium-starved cells stained with DCFD/PI was performed by time-lapse microscopy. Cells were shifted in SCD_{Cd} medium at time 0 and DCFDA and PI fluorescence was monitored every 15 min for about 10 hours.
